# Cognitive and cortical network alterations in pediatric temporal lobe space-occupying lesions: an fMRI study

**DOI:** 10.3389/fnhum.2024.1509899

**Published:** 2024-12-09

**Authors:** Bohan Hu, Xueyi Guan, Huina Zhai, Xu Han, Cuiling Hu, Jian Gong

**Affiliations:** ^1^Department of Pediatric Neurosurgery, Beijing Tiantan Hospital, Capital Medical University, Beijing, China; ^2^Beijing Neurosurgical Institute, Capital Medical University, Beijing, China; ^3^Hefei Comprehensive National Science Center, Institute of Artificial Intelligence, Hefei, Anhui, China; ^4^Beijing RIMAG Medical Imaging Center, Beijing, China

**Keywords:** pediatric, cognition, cerebrum network, ineffective compensation, prehabilitation, default mode network (DMN)

## Abstract

**Background:**

Temporal lobe mass lesions are the most common intracranial space-occupying lesions in children, among various brain lobes. The temporal lobe is critically involved in higher cognitive functions, and surgical interventions often risk causing damage to these functions. If necessary interventions and prehabilitation can be conducted preoperatively, it might be possible to achieve a larger extent of lesion resection with minimal cognitive impairment. However, research in this area has been relatively limited in the past. Our study aims to fill this gap.

**Methods:**

We enrolled 15 children with temporal lobe mass lesions and 15 age- and gender-matched healthy children as controls. All participants underwent cognitive assessments and functional MRI scans. The cognitive testing data and functional MRI data were then analyzed and compared between the two groups.

**Results:**

Our findings suggest that children with temporal lobe mass lesions primarily exhibit impairments in working memory and sustained attention. Multiple brain network indices were altered in the affected children, with the most prominent change being hyperactivation of the default mode network (DMN). This hyperactivation was correlated with cognitive impairments, indicating that the overactivation of the DMN might represent an inefficient compensatory mechanism within the brain’s networks.

**Conclusion:**

Compared to healthy children, those with temporal lobe mass lesions experience deficits in working memory and sustained attention, and the hyperactivation of the DMN may be the underlying network mechanism driving these cognitive impairments. Our research offers a unique and clinically valuable reference for future studies on preoperative interventions and prehabilitation in this population.

## 1 Introduction

Intracranial space-occupying lesions (ISOLs) primarily occur in the brain and central nervous system and are common in children, representing the most prevalent type of solid tumor in this age group (Ostrom et al., 2021). The temporal lobe is the most frequently affected site within the pediatric brain lobes (Ostrom et al., 2021). The functions of the temporal lobe are mainly associated with higher-order cognitive processes, such as memory (Preston and Wagner, 2007), auditory (Steinschneider et al., 2013) and language (Visser et al., 2010). The majority of pediatric ISOLs are classified as low- to intermediate-grade or benign (Cohen, 2022), with surgical resection typically sufficient to achieve favorable long-term outcomes and survival, often without the need for further treatment. Nonetheless, postoperative cognitive impairments are frequently unavoidable (Vaz, 2004; Sherman et al., 2011; Lambon Ralph et al., 2012). Although optimizing surgical techniques and carefully adjusting the extent of resection may improve cognitive outcomes (Berg, 2015), implementing preoperative interventions could potentially enable more extensive lesion resection while minimizing cognitive deficits (Sughrue et al., 2022). Previous studies have demonstrated that invasive or non-invasive brain stimulation techniques can facilitate the reorganization of higher-order cognitive networks, thereby enabling complete tumor resection without resulting in permanent complications (Duffau et al., 2002; Duffau, 2014; Hamer and Yeo, 2022; Sughrue et al., 2022). Therefore, investigating the alterations in brain networks and cognitive functions caused by lesions may offer valuable insights for the prehabilitation of pediatric patients with temporal lobe space-occupying lesions.

Resting-state functional magnetic resonance imaging (rs-fMRI) provides a non-invasive approach to explore brain activity and functional networks, offering insights into the cognitive changes and neural mechanisms associated with brain disorders (Stam, 2014). Current research on brain network alterations in ISOLs primarily focuses on adult populations (Ghumman et al., 2016; Jutten et al., 2020; Tordjman et al., 2021), while studies involving pediatric patients remain limited. Some investigations have examined pediatric tumor survivors (Zhang et al., 2008; Robinson et al., 2014), while others have focused on the impact of surgical injury itself on cognition and brain networks (Guan et al., 2022; Guan et al., 2024b). Additionally, there are studies on traumatic brain injury in children and adolescents (Keightley et al., 2011; Tlustos et al., 2015; Tuerk et al., 2020), as well as research on perinatal stroke in pediatric populations (Saunders et al., 2019). However, studies specifically addressing brain network and cognitive alterations caused by isolated ISOLs are scarce. Our research aims to fill this gap.

We conducted a comparative analysis of rs-fMRI metrics and cognitive functions between children with temporal lobe space-occupying lesions and typically developing children to identify group differences in brain functional networks and higher-order cognitive abilities. By elucidating these intergroup differences, we aim to gain a deeper understanding of the impact of these lesions on pediatric cognitive function and the underlying neural network mechanisms. These findings are expected to provide unique and clinically valuable insights for the design and implementation of preoperative interventions and personalized prehabilitation strategies in the future.

## 2 Materials and methods

### 2.1 Participants

The samples included in this study consist of preoperative pediatric patients and age- and gender-matched healthy children, with all patient data collected prior to surgery. We enrolled 15 pediatric patients with temporal lobe lesions as the patient group, who were treated at the Pediatric Neurosurgery Department of Beijing Tiantan Hospital between November 2021 and January 2024. All 15 patients underwent preoperative fMRI scans. However, due to non-compliance in completing cognitive assessments by two participants, cognitive data were only obtained from 13 children.

We also recruited 15 age- and gender-matched healthy children as the control group from Beijing RIMAG Medical Imaging Center, who underwent the same MRI scans and cognitive evaluations.

For the patient group, the inclusion criteria were: willingness to participate in the study; age between 6 and 18 years; diagnosed with a primary temporal lobe space-occupying lesion requiring surgical intervention. The exclusion criteria were: withdrawal from the study for any reason; refusal to cooperate in completing the MRI scan; presence of medical conditions affecting the brain; or contraindications to anesthesia.

For the control group, the inclusion criteria were: willingness to cooperate in completing MRI scans and cognitive assessments; age between 6 and 18 years; no history of brain disease. The exclusion criteria were: withdrawal from the study for any reason; inability to cooperate in completing the MRI scans or cognitive tests.

### 2.2 Ethical statements

Parental written informed consent was collected for all children who participated in the study. The research adhered to the ethical guidelines outlined in the Declaration of Helsinki and received approval from Institutional Review Board of Beijing Tiantan Hospital, Capital Medical University (KY 2021-100-02).

### 2.3 Cognitive assessment

Each participant’s neurocognitive abilities were assessed using the CNS Vital Signs (CNS VS) battery, a digital tool designed for repeated assessments and widely recognized for its reliability and validity (Gualtieri and Johnson, 2006). This 30–40 min assessment evaluates 15 cognitive domains, providing age-adjusted scores based on 10 subtests. The cognitive domains measured include Composite Memory (CM), Verbal Memory (VerbM), Visual Memory (VisM), Psychomotor Speed (PsyMotSpd), Reaction Time (RT), Complex Attention (ComA), Cognitive Flexibility (CogFlex), Processing Speed (ProcSpd), Executive Function (ExeFun), Social Acuity (SocAcu), Reasoning (Reason), Working Memory (WM), Sustained Attention (SustA), Simple Attention (SimA), and Motor Speed (MotSpd). Additionally, the Neurocognitive Index (NCI), derived from CM, PsyMotSpd, RT, CogFlex, and ComA, provides an overall measure of brain function. These assessments were conducted before surgery, in the same week as neuroimaging. For the children in the control group, cognitive testing and imaging were completed on the same day.

### 2.4 Imaging data acquisition

The participants were directed to maintain a seated position while keeping their eyes shut. Administration of sedatives was omitted throughout the examination process. All patients and healthy controls underwent MRI scans on a 3T scanner (MAGNETOM Prisma, Siemens Healthcare, Erlangen, Germany) with a 64-channel head/neck coil. The software version is Syngo MR E11. The protocol included T1 weighted structure imaging with magnetization prepared rapid acquisition gradient echo (MPRAGE) sequence and rs-fMRI with an echo-planar imaging (EPI) sequence. The scan parameters for the MPRAGE sequence were: repetition time (TR) = 1560 ms; echo time (TE) = 1.65 ms; flip angle = 8°; slices = 176; field of view (FOV) = 256 × 256 mm; and voxel size = 1 mm isotropic. The parameters for EPI with simultaneous multislice (SMS) acceleration technique were: TR = 2000 ms; TE = 35 ms; slices = 69; SMS = 3; FOV = 207 × 207 mm; voxel size = 2.2 mm isotropic; volumes = 240.

The imaging data for the patients enrolled in the study were obtained through scans conducted at Beijing Tiantan Hospital. The imaging data for the normal controls were collected at Beijing RIMAG Medical Imaging Center. The MRI equipment used at both locations was of the identical model, and the scanning parameters and software version employed were exactly the same.

### 2.5 Imaging data preprocessing

The preprocessing of rs-fMRI images was performed using the Data Processing and Analysis of Brain Imaging toolbox (Chao-Gan and Yu-Feng, 2010; Yan et al., 2016) (DPABI v6.2)^1^ and Statistical Parametric Mapping software (SPM 12),^2^ both operating within MATLAB (Matlab Release 2020b, Mathworks Inc., Natick, MA). The following steps were included in the preprocessing workflow:

(a) The initial 10 images were discarded; (b) Slice-timing and head motion corrections were applied. Participants with head motion exceeding 3 mm in translation or 3° in rotation were excluded from analysis, and one patient was removed due to excessive motion during the MRI scan; (c) Linear trends, cerebrospinal fluid signals, white matter, and the Friston 24-parameter model of head motion were regressed as nuisance covariates from the blood oxygen-level-dependent (BOLD) signal. Global signal regression was not employed in this process (He and Liu, 2012; Saad et al., 2012); (d) Functional images were normalized to the Montreal Neurological Institute (MNI) space using DARTEL (Ashburner, 2007) and resampled to a 3 mm cubic voxel size; (e) Band-pass filtering was performed in the range of 0.01 Hz to 0.10 Hz; (f) The images were smoothed with a full width at half maximum of 4 × 4 × 4 mm.

**FIGURE 1:**
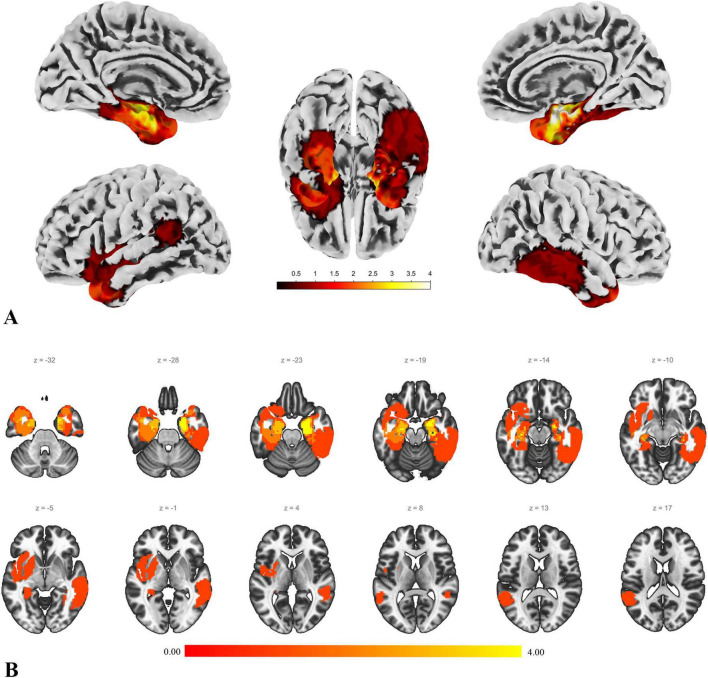
Lesion distribution. This figure illustrates the lesion distribution of the enrolled patients. **(A)** The result of projecting the voxel map of lesion distribution onto the cortical surface, providing a more intuitive view of the lesion distribution. The color intensity represents the degree of overlap in lesion distribution. **(B)** A slice view of the lesion distribution. The figure shows that some lesions involve the basal ganglia and parts of the orbitofrontal region. However, the actual lesions compress these areas, and after normalization, they are displayed in the state shown in the figure.

The data from the patient group and the control group were preprocessed separately. Additionally, to achieve more accurate normalization, the lesion areas in the T1 images of the patient group were manually removed by an experienced neurosurgeon (Rorden et al., 2007; Andersen et al., 2010; Guan et al., 2022). Figure 1 illustrates the lesion distribution for all enrolled patients.

### 2.6 Brain imaging data analysis

Commonly used metrics and methods in brain network analysis include those that reflect local brain function changes (Lv et al., 2018), such as Regional Homogeneity (ReHo) (Zang et al., 2004), Amplitude of Low Frequency Fluctuations (ALFF) (Zang et al., 2007), and Fractional ALFF (fALFF) (Zou et al., 2008). Additionally, functional connectivity (FC) analysis is employed to assess the functional coordination between predefined brain regions or between predefined regions and other voxels (Lv et al., 2018).

In this study, we calculated ReHo, ALFF, and fALFF to assess local brain function changes. For whole-brain FC analysis, we predefined seed points (i.e., regions of interest, ROI) based on the results of ReHo, ALFF, and fALFF. Seed-based analysis (SBA) was then conducted using these predefined regions.

The method for defining the seed points involved extracting the overlapping brain regions that showed significant group differences across the three metrics—ReHo, ALFF, and fALFF. The regions where patients had higher values than the control group and the regions where patients had lower values were extracted separately. Among the identified overlapping brain regions, any region with fewer than 19 voxels was excluded from being used as a seed point for subsequent SBA analysis. The threshold of 19 voxels was set because a region of interest (ROI) is typically defined as a sphere with a 5 mm radius (Creem and Proffitt, 2001) or 6 mm (DeSimone et al., 2021). A sphere with a 5mm radius occupies 19 voxels in an image with a resolution of 3 mm.

The localization of nodes and clusters was identified using the Automated Anatomical Labeling (AAL) atlas (Tzourio-Mazoyer et al., 2002). Additionally, we applied the “winner-takes-all” approach to map the AAL brain regions onto Yeo’s 7-network model (Yeo et al., 2011). Detailed methods and the correspondence between regions and networks can be found in Supplementary Materials 1, 2 for further reference.

### 2.7 Exploratory correlation analysis

We conducted Spearman rank correlation analyses between the cognitive domains and imaging markers that showed significant differences between the patient and control groups. Due to missing cognitive test data for two patients, the exploratory correlation analysis included 13 patients and 15 controls. The specific items included in the correlation analysis are detailed in the section “3 Results.”

### 2.8 Statistical analysis and result display

In this study, independent samples *t*-tests were applied to continuous variables (cognitive tests, age, imaging data), while chi-square tests were used for categorical variables (gender). The statistical analyses for cognitive tests, age, and gender were conducted using SPSS (IBM SPSS Statistics version 26, IBM Corporation), with a significance level set at 0.05 (two-tailed). Given the exploratory nature of the study, no multiple comparisons correction was applied to the cognitive test data (Guan et al., 2022; Guan et al., 2024a).

For imaging data, after performing independent samples *t*-tests, multiple comparisons correction was necessary. We applied Gaussian random-field theory (GRF) correction (voxel-level *p* < 0.001, cluster-level *p* < 0.05, two-tailed) provided in the DPABI software (Chao-Gan and Yu-Feng, 2010; Yan et al., 2016). Additionally, to validate the robustness of our findings, we applied false discovery rate (FDR) correction to the imaging metrics, setting the overall significance level at 0.05. Based on previous studies, FDR correction typically involves setting a voxel threshold, often at 20 voxels (Lieberman and Cunningham, 2009). In this study, we selected a threshold of 50 voxels to further assess the robustness of our results. It is also important to note that all our statistical analyses and multiple comparison corrections were conducted exclusively on unaffected cortical regions. Lesion-affected areas, subcortical structures and the cerebellum were not included within the scope of this study’s analysis.

For the exploratory correlational analysis, given its exploratory nature, we primarily present and interpret results without multiple comparison corrections (Xu et al., 2022; Guan et al., 2024a). However, we have also provided the results adjusted using FDR correction as Supplementary Information, offering readers additional insights. The statistical significance level was set at 0.05 (two-tailed).

Data visualization was carried out using BrainNet Viewer (Xia et al., 2013), Computational Anatomy Toolbox 12,^3^ CONN’s visualization tools (Whitfield-Gabrieli and Nieto-Castanon, 2012) and GraphPad 8.

## 3 Results

### 3.1 Demographics of enrolled subjects

A total of 30 participants were enrolled in this study, including 15 pediatric patients and 15 age- and gender-matched healthy controls (with no significant differences in age or gender). The mean age was approximately 10 years, with an equal gender distribution (8 males and 7 females). In the patient group, two-thirds presented with epilepsy as the initial symptom. Tumors accounted for 80% of the cases, with the majority being low-grade lesions. The distribution of lesion laterality was roughly balanced (7 left-sided and 8 right-sided), and the average lesion volume was 16.5 cm^3^. Detailed demographic information is provided in Table 1, and the histopathological diagnoses for each enrolled patient are presented in Supplementary Material 3.

**TABLE 1:** Demographics of enrolled subjects.

	Patient group (*n* = 15)	Control group (*n* = 15)	*p*-value
Age (mean ± SD)^#^	10.6 ± 2.6	10.9 ± 2.8	0.74
Gender (male: female)	8:7	8:7	1
Lesion side (left: right)	7:8	–	
Initial symptom (seizure: headache: diplopia: asymptomatic)	10:1:1:3	–	
Lesion volume (mean ± SD, mm^3^)*	16548.7 ± 25834.6	–	
Lesion type (Tumor: Vascular malformation: others)	12:2:1	–	

#Patients’ age ranges from 6.0 to 15.4; controls’ age ranges from 7.0 to 16.3.

*Lesion volume ranges from 153.9 to 98187.8 mm^3^.

### 3.2 Cognitive assessment

**FIGURE 2:**
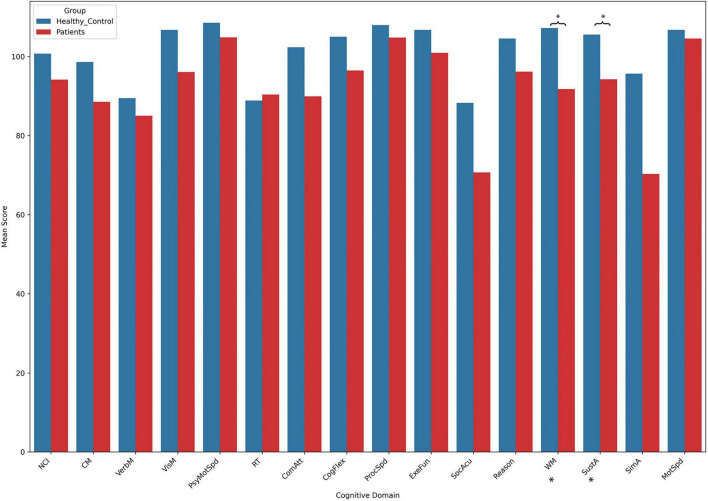
The results of cognitive assessment. This bar chart illustrates the mean scores across different cognitive domains between the two groups. Asterisks (*) indicate statistically significant group differences. Detailed *t*- and *p*-values can be found in Table 2.

The results of the cognitive tests revealed no significant differences between the patient group and the control group across most cognitive domains. However, the patient group showed significantly lower scores in two specific areas: WM and SustA. In this study, WM reflects the ability to perceive and attend to symbols during short-term memory tasks, while SustA indicates the ability to guide and focus cognitive activity on specific stimuli. These findings suggest that certain cognitive domains are significantly impaired in children with temporal lobe lesions, though most cognitive functions remain relatively intact. The detailed information is presented in Figure 2 and Table 2. Subsequent analyses and result discussions were also primarily focused on these two cognitive domains. Here, we would like to elaborate further on the rationale for selecting SustA and WM. Firstly, these two cognitive domains exhibited significant differences among various cognitive areas. Given the limited prior knowledge available for reference, our study is inherently exploratory. Focusing on cognitive domains with evident differences may enhance the efficiency of our research. Secondly, previous studies involving adult patients have shown that the temporal lobe functions are indeed associated with sustained attention and memory (Vaz, 2004; Zhang et al., 2009; Lambon Ralph et al., 2012; Bauman et al., 2019). This connection further rationalizes our selection of these two cognitive domains and indirectly supports the findings of our study.

**TABLE 2:** Cognitive results between patients and controls.

	Patients’ mean (*n* = 13)^#^	Controls’ mean (*n* = 15)	*t*-value	*p*-value
Neurocognition index	94.2	100.7	−1.0	0.329
Composite memory	88.5	98.6	−0.9	0.374
Verbal memory	85.0	89.5	−0.4	0.721
Visual memory	96.1	106.7	−1.3	0.213
Psychomotor speed	104.9	108.5	−0.6	0.569
Reaction time	90.4	88.9	0.2	0.862
Complex attention	89.9	102.3	−1.6	0.117
Cognitive flexibility	96.5	105.0	−1.1	0.270
Processing speed	104.8	107.9	−0.5	0.605
Executive function	100.9	106.7	−0.8	0.409
Social acuity	70.7	88.3	−1.5	0.158
Reasoning	96.2	104.5	−1.4	0.165
Working memory*	91.8	107.2	−2.1	0.042
Sustained attention*	94.2	105.5	−2.3	0.032
Simple attention	70.3	95.7	−1.1	0.300
Motor speed	104.5	106.7	−0.4	0.720

#Due to the non-cooperation of two pediatric patients in the testing process, data for these two patients are consequently unavailable.

*Represents significant differences between the patient group and the control group.

### 3.3 Brain imaging analysis

***ALFF, fALFF and Reho***

ALFF reflects the overall activity level of a specific brain region during the resting state, with higher ALFF values typically indicating stronger activity in that region (Zang et al., 2007). fALFF offers a more precise measure, focusing more specifically on low-frequency fluctuations related to brain function, and is generally better at distinguishing functional brain regions from non-functional areas (such as noise) (Zou et al., 2008). ReHo measures whether the neuronal activity in a given region is synchronized with its surrounding voxels, or in other words, whether these regions are working together (Zang et al., 2004). All three metrics are capable of reflecting local changes in brain function.

**FIGURE 3:**
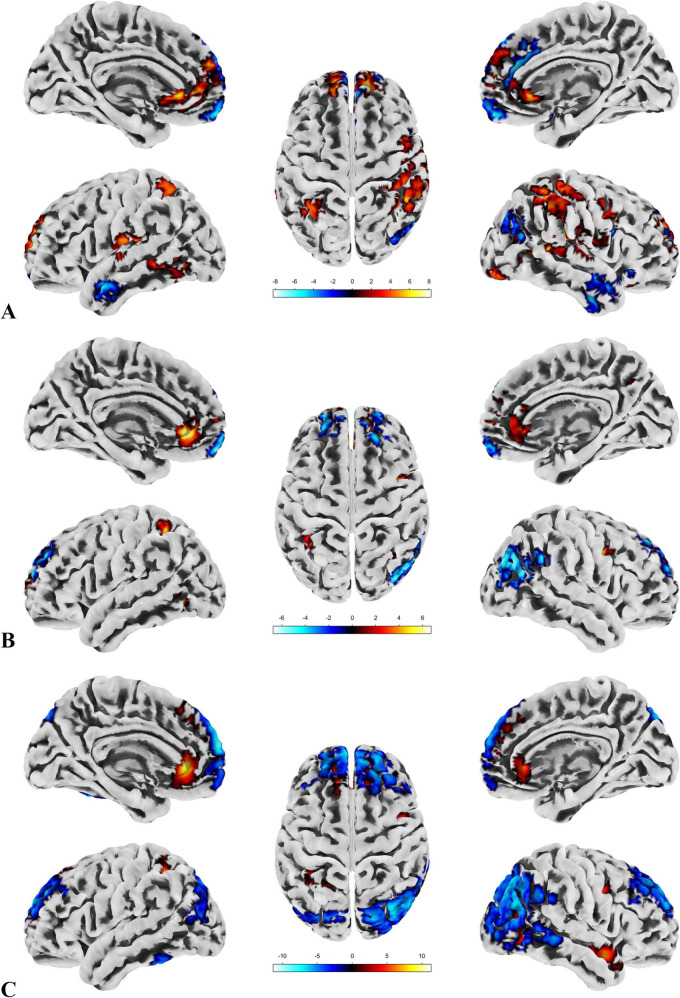
ALFF, fALFF and Reho. **(A)** Highlights the brain regions with significant differences in ALFF between the patient and control groups. **(B)** Depicts the brain regions where significant differences in fALFF were observed between the patient and control groups. **(C)** Displays the brain regions showing significant differences in ReHo between the patient and control groups. A direct observation reveals a certain degree of similarity in the distribution patterns of the significant brain regions across the three metrics.

**FIGURE 4:**
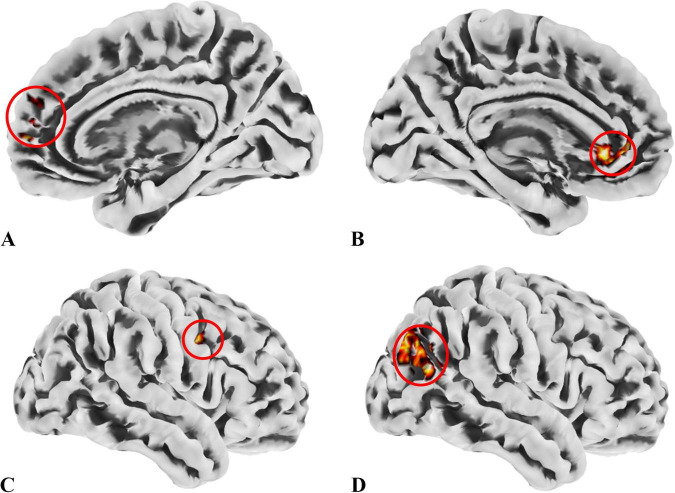
ROIs identified based on ALFF, fALFF, and ReHo metrics. **(A)** ROI1, anatomically labeled as Frontal_Sup_Medial_R; **(B)** ROI2, anatomically labeled as Cingulum_Ant_L; **(C)** ROI3, anatomically labeled as Precentral_R; **(D)** ROI4, anatomically labeled as Occipital_Mid_R; ROIs 1–3 were identified from regions where the patient group showed higher values across all three metrics than the control group, while ROI4 was derived from regions with lower values in the patient group. The colors in the figure are solely for the purpose of identifying anatomical locations and serve no other function.

In the patient group, compared to the control group, some brain regions exhibited higher values, while others showed lower values across all three metrics. For ALFF, we identified 18 clusters with significant differences (Figure 3A), and the detailed coordinates and anatomical labels are listed in Table 3. For fALFF and ReHo, we found 12 clusters each (Figures 3B, C), with the detailed information provided in Tables 4, 5, respectively. By observing Figure 3, we can see a certain degree of similarity in the distribution patterns across these three metrics. We predefined the seed points for SBA analysis based on the three metrics. Brain regions where the patient group showed higher values than the control group and regions where they showed lower values were separately aggregated to identify common regions. The coordinates and anatomical labels of these regions were determined by selecting the voxel with the highest absolute sum of the values across the three metrics in each overlapping region. This method of defining ROIs was chosen because, as mentioned earlier, the three metrics share certain similarities and are dimensionless. Thus, we hypothesize that if a brain region shows significant differences across all three metrics with a consistent trend, it may represent an important area deserving further attention. Consequently, the voxel with the highest absolute summed value across the three metrics was selected to represent each cluster. Ultimately, we identified four ROIs for subsequent SBA (Figure 4 and Table 6). ROI 1–3 were defined based on the overlapping brain regions where the patient group exhibited higher values across all three metrics compared to the control group. ROI4 was identified based on the overlapping brain region where the patient group showed lower values across all three metrics relative to the control group.

**TABLE 3:** The results of ALFF.

Cluster no.	Peak coordinate (X, Y, Z)	Peak intensity	Peak label (AAL)	Yeo’s network	Cluster size (voxels)
Cluster 1	45, −27, 21	8.5	Rolandic_Oper_R	Sensorimotor network	266
Cluster 2	15, 63, 18	9.9	Frontal_Sup_R	Dorsal attention	257
Cluster 3	3, 54, 48	−9.1	Frontal_Sup_Medial_R	Default mode network	241
Cluster 4	48, 3, −9	−7.7	Temporal_Sup_R	Sensorimotor network	163
Cluster 5	−3, 30, −3	7.5	Cingulum_Ant_L	Default mode network	108
Cluster 6	48, 12, 24	−6.2	Frontal_Inf_Oper_R	Frontoparietal network	86
Cluster 7	−54, 9, −24	−9.1	Temporal_Inf_L	Limbic	62
Cluster 8	−39, −57, 60	5.5	Parietal_Inf_L	Frontoparietal network	60
Cluster 9	−45, −57, −12	5.6	Occipital_Inf_L	Visual network	59
Cluster 10	−3, 63, −21	−6.5	Rectus_L	Limbic	56
Cluster 11	−66, −18, 18	5.6	Postcentral_L	Sensorimotor network	48
Cluster 12	39, −66, 21	6.9	Temporal_Mid_R	Default mode network	45
Cluster 13	39, 33, −6	−6.2	Frontal_Inf_Orb_R	Default mode network	41
Cluster 14	57, −69, 21	−6.8	Temporal_Mid_R	Default mode network	37
Cluster 15	60, −15, 27	5.1	SupraMarginal_R	Ventral attention	29
Cluster 16	−51, −48, −6	5.8	Temporal_Inf_L	Limbic	28
Cluster 17	24, 27, −21	6.6	Frontal_Inf_Orb_R	Default mode network	24
Cluster 18	33, −90, −12	5.8	Occipital_Inf_R	Visual network	22

**TABLE 4:** The results of fALFF.

Cluster no.	Peak coordinate (X, Y, Z)	Peak intensity	Peak label (AAL)	Yeo’s network	Cluster size (voxels)
Cluster 1	−6, 39, −9	7.0	Frontal_Med_Orb_L	Default mode network	117
Cluster 2	48, −75, 30	−7.1	Occipital_Mid_R	Visual network	113
Cluster 3	24, 51, 48	−6.1	Frontal_Sup_R	Dorsal attention	82
Cluster 4	−27, 66, 21	−5.8	Frontal_Sup_L	Dorsal attention	66
Cluster 5	−36, −51, 60	5.6	Parietal_Sup_L	Dorsal attention	63
Cluster 6	12, 57, 21	6.5	Frontal_Sup_Medial_R	Default mode network	52
Cluster 7	6, 66, −18	−7.0	Frontal_Sup_Orb_R	Limbic	39
Cluster 8	12, −63, 36	6.3	Precuneus_R	Default mode network	34
Cluster 9	42, 3, 30	6.9	Precentral_R	Sensorimotor network	34
Cluster 10	−15, 63, 9	6.4	Frontal_Sup_L	Dorsal attention	30
Cluster 11	−45, −66, −3	5.7	Occipital_Inf_L	Visual network	24
Cluster 12	30, −60, 42	6.1	Angular_R	Default mode network	23

**TABLE 5:** The results of Reho.

Cluster no.	Peak coordinate (X, Y, Z)	Peak intensity	Peak label (AAL)	Yeo’s network	Cluster size (voxels)
Cluster 1	−3, 72, 21	−10.1	Frontal_Sup_Medial_L	Default mode network	949
Cluster 2	39, −81, 42	−7.9	Occipital_Mid_R	Visual network	498
Cluster 3	−6, 36, 3	12.2	Cingulum_Ant_L	Default mode network	148
Cluster 4	−24, −84, 45	−6.2	Occipital_Sup_L	Visual network	103
Cluster 5	−36, −51, 57	7.6	Parietal_Inf_L	Frontoparietal network	77
Cluster 6	39, 3, 36	7.8	Precentral_R	Sensorimotor network	75
Cluster 7	45, −69, −3	5.7	Temporal_Inf_R	Limbic	67
Cluster 8	60, −63, −6	−6.1	Temporal_Inf_R	Limbic	61
Cluster 9	−12, 36, 48	7.2	Frontal_Sup_L	Dorsal attention	58
Cluster 10	51, 6, −18	7.0	Temporal_Pole_Mid_R	Default mode network	56
Cluster 11	−51, −45, −27	−7.2	Temporal_Inf_L	Limbic	54
Cluster 12	−21, 54, −6	5.6	Frontal_Sup_Orb_L	Limbic	47

**TABLE 6:** Brain regions overlapping across ALFF, fALFF, and ReHo metrics for subsequent SBA.

ROI no.	Coordinate (X, Y, Z)	Label (AAL)	Yeo’s network	Cluster size (voxels)
ROI 1	12, 57, 21	Frontal_Sup_Medial_R	Default mode network	42
ROI 2	−6, 33, 0	Cingulum_Ant_L	Default mode network	26
ROI 3	42, 3, 30	Precentral_R	Sensorimotor network	21
ROI 4	51, −75, 27	Occipital_Mid_R	Visual network	35

***SBA***

**FIGURE 5:**
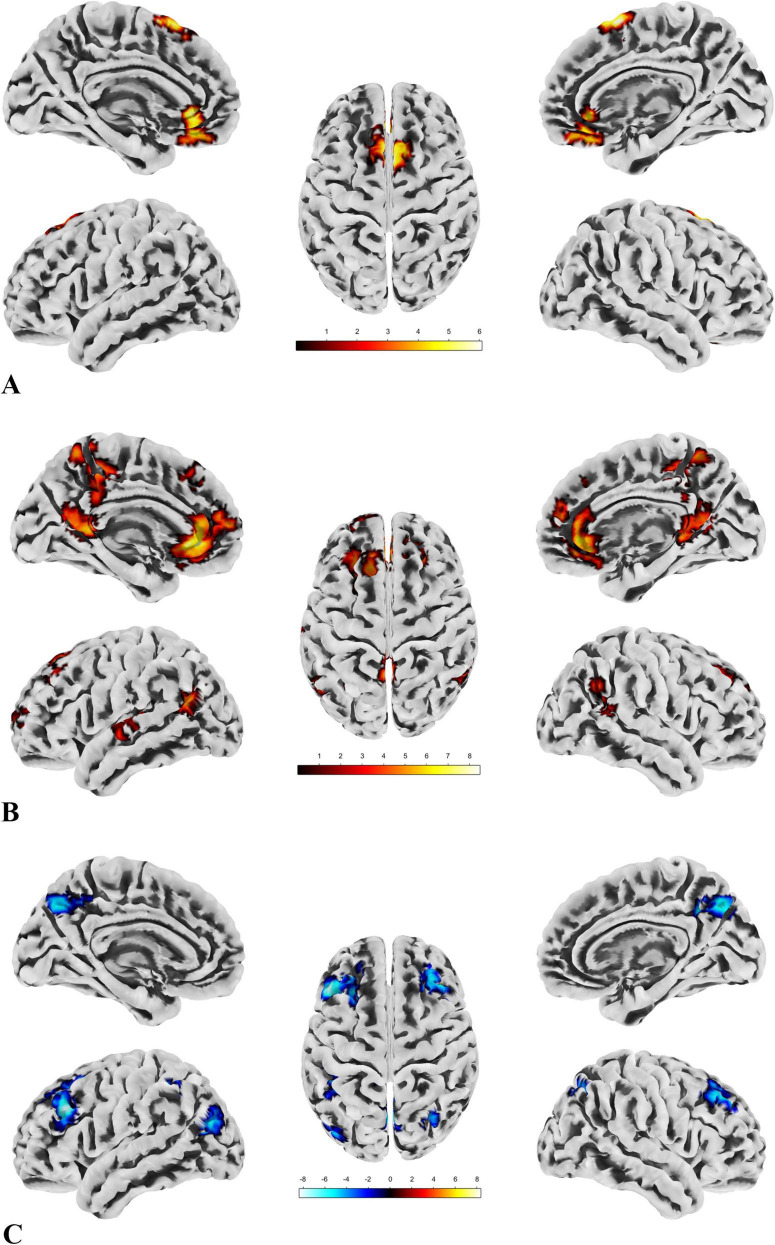
The results of SBA. **(A)** There are two clusters with significant group differences in FC with ROI1, both showing greater connectivity in the patient group compared to the control group. The distribution of the ROI and clusters is primarily located in the DMN and sensorimotor network. **(B)** Seven clusters show significant group differences in FC with ROI2, all with greater connectivity in the patient group compared to the control group. The ROI and clusters are mainly distributed across the DMN, sensorimotor network, and visual network. **(C)** Six clusters exhibit significant group differences in FC with ROI4, all showing reduced connectivity in the patient group compared to the control group. The ROI and clusters are primarily located in the visual network, DMN, and frontoparietal network.

We conducted SBA using the four ROIs determined by the local brain function metrics and found significant differences only in ROI1, ROI2, and ROI4. This indicates that functional connectivity between certain brain regions and these ROIs differed between the groups. The results are presented in Figure 5, with detailed information provided in Table 7.

**TABLE 7:** The results of SBA.

ROI	ROI coordinate (x, y, z)	ROI label	Yeo’s network of ROIs	Significant brain cluster no.	Peak coordinate (x, y, z)	peak intensity	Peak label	Cluster size (voxels)	Yeo’s network of clusters
ROI 1	12, 57, 21	Frontal_Sup_Medial_R	Default mode network	Cluster 1	−6, 36, −3	5.5	Cingulum_Ant_L	123	Default mode network
				Cluster 2	3, 12, 66	6.9	Supp_Motor_Area_R	110	Sensorimotor network
ROI 2	−6, 33, 0	Cingulum_Ant_L	Default mode network	Cluster 1	−9, 36, −3	9.2	Cingulum_Ant_L	463	Default mode network
				Cluster 2	−6, −45, 6	5.9	Calcarine_L	198	Visual network
				Cluster 3	−12, 33, 51	6.3	Frontal_Sup_L	174	Dorsal attention
				Cluster 4	0, −54, 63	5.7	Precuneus_L	151	Default mode network
				Cluster 5	−48, −63, 21	5.4	Temporal_Mid_L	109	Default mode network
				Cluster 6	48, −54, 21	5.2	Temporal_Mid_R	78	Default mode network
				Cluster 7	−45, −27, 15	4.3	Rolandic_Oper_L	65	Sensorimotor network
ROI 4	51, −75, 27	Occipital_Mid_R	Visual network	Cluster 1	−48, 33, 36	−8.6	Frontal_Mid_L	159	Frontoparietal network
				Cluster 2	15, −57, 39	−6.2	Precuneus_R	156	Default mode network
				Cluster 3	−48, −39, 39	−5.5	Parietal_Inf_L	89	Frontoparietal network
				Cluster 4	39, 36, 45	−6.9	Frontal_Mid_R	82	Frontoparietal network
				Cluster 5	−45, −81, 24	−6.1	Occipital_Mid_L	50	Visual network
				Cluster 6	30, −69, 42	−4.7	Occipital_Sup_R	49	Visual network

For all imaging data results, we have provided the full details, such as the labels of clusters spanning multiple brain regions, in the Supplementary Material 4 for verification. Additionally, among the metrics applied in this study, the spatial distribution and trend of brain regions passing FDR correction closely resemble those passing GRF correction, with the exception of ROI 1 in the SBA, which did not survive correction. This suggests that the enhanced FC between ROI 1 and the left anterior cingulate cortex as well as the right supplementary motor area may lack stability. However, this does not affect the overall trend or interpretation of our key findings. Detailed results following FDR correction, including visualizations of significant brain regions and their distribution and trends, are provided in Supplementary Material 4 for readers’ reference.

### 3.4 Exploratory correlation analysis

**FIGURE 6:**
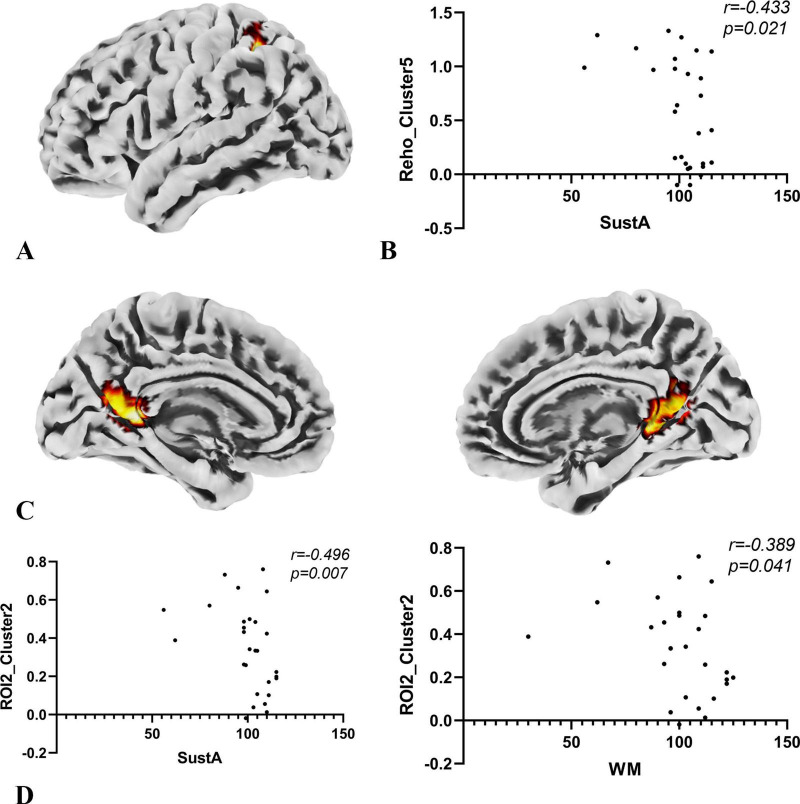
Results of correlation analysis. **(A)** The anatomical location of cluster 5 in the ReHo metric, with its peak point anatomically labeled as Parietal_Inf_L. The color of the cluster was used only to suggest anatomical location. **(B)** Spearman correlation analysis indicates a negative correlation between the SuStA domain and ReHo cluster 5. **(C)** In the SBA analysis, brain regions showing significant differences with ROI2 are distributed across both hemispheres, as depicted in the figure; however, the peak is located on the left, specifically at Calcarine_L. The color of the clusters was used only to suggest anatomical location. **(D)** Both SustA and WM exhibit negative correlation with the FC value of ROI2 and cluster 2.

In the exploratory Spearman rank correlation analysis, we found that the two cognitive domains, SustA and WM, which showed significant group differences, were correlated with imaging metrics. There was a negative correlation between SustA and ReHo cluster 5, and both SustA and WM showed negative correlation with the FC value between ROI2 and Cluster 2. The detailed results, including correlation coefficients and *p*-values, are presented in Figure 6. We observed that the correlation analysis primarily involves the frontoparietal network, default mode network, and visual network. Additionally, none of the results from the correlational analysis remained significant after FDR correction. This information has been included in Supplementary Material 5.

## 4 Discussion

This study investigates the patterns of cognitive and cortical brain network alterations in children with temporal lobe lesions compared to typically developing children. Our findings reveal significant reductions in WM and SustA in affected children, alongside widespread cortical brain network changes, characterized primarily by hyperactivation of the default mode network (DMN). The results of correlation analyses suggest that there may be ineffective compensation.

The decline in WM and SustA in the cognitive domain is consistent with numerous previous studies. Memory impairment is a well-documented symptom in patients with Alzheimer’s disease, particularly those with onset in the medial temporal lobe (Dickerson and Sperling, 2008). Similarly, patients with temporal lobe epilepsy have also been reported to experience deficits in both memory and attention (Zhang et al., 2009; Cataldi et al., 2013; Celiker Uslu et al., 2019). The phenomenon of DMN hyperactivation accompanied by extensive changes in other brain networks has been reported in previous studies and interpreted as a compensatory mechanism at the network level (Bonnelle et al., 2011; Chen et al., 2016; Tordjman et al., 2021). In other words, this represents an objective alteration in the DMN and other brain network itself, when confronted with external perturbations.

Our correlation analysis found a negative correlation between the ReHo values in the left inferior parietal lobule (IPL), a region in the frontoparietal network, and SustA. Additionally, FC values between the left anterior cingulate cortex (ACC), left calcarine sulcus, and bilateral precuneus (within the DMN and between the DMN and visual networks) were negatively correlated with both WM and SustA. This negative correlation suggests the potential presence of inefficient compensatory mechanisms in the cortical functional networks of these children with temporal lobe lesions (Shumskaya et al., 2017; Simos et al., 2023; Hong et al., 2024).

Previous studies have shown that the parietal lobe is involved in attentional processes (Lawrence et al., 2003; Behrmann et al., 2004). Clower et al. (2001) demonstrated that the IPL plays a role in mechanisms of eye movement and attention, supported by subcortical systems such as the hippocampus. In patients with temporal lobe lesions, where structures like the temporal lobe and hippocampus are damaged, the overactivation of the IPL may represent a compensatory mechanism. A study on sustained attention in patients with stable bipolar disorder and their unaffected relatives revealed that during high task loads, activation in the left insula and bilateral IPL significantly increased in the relatives (Sepede et al., 2012). This suggests that these individuals may compensate for sustained attention deficits by increasing activation in these regions. Our findings align with this, suggesting that IPL activation might represent a compensatory mechanism at the brain network level. However, this compensation appears ineffective (Simos et al., 2023), as SustA decreases with increased IPL activation. Rosenberg et al. (2016) found that activation in temporal and parietal regions was associated with decreased attentional performance in their study on the sustained attention network, which may also help explain our results.

The ACC serves as a critical hub for information processing and regulation within the brain, playing a key role in conflict monitoring, error detection, and decision-making (Gehring and Fencsik, 2001; Botvinick et al., 2004; Margulies et al., 2007). It is also involved in attention and memory processes (Lenartowicz and McIntosh, 2005). The DMN is a highly robust resting-state brain network (Buckner et al., 2008). In a study examining the effects of tumors on the DMN, researchers found that the DMN exhibited resilience to the presence of tumors, with most brain regions showing little to no impact on its functioning (Ghumman et al., 2016). In another study investigating the effects of temporal lobe epilepsy on the DMN, researchers observed an increase in FC in the posterior cingulate cortex, which was interpreted as a potential compensatory mechanism of the brain (Zhang et al., 2010). These studies may suggest that the DMN may be more likely to remain stable or activated when the brain is injured.

In our study, we observed increased FC between the ACC and regions near the calcarine sulcus, including the precuneus, in the patient group. This increased connectivity showed a negative correlation with both SustA and WM. This may represent a compensatory effect within the brain network; however, such compensation may be ineffective. A study on schizophrenia patients and their relatives found abnormally high FC within the DMN both at rest and during tasks, closely associated with psychopathology (Whitfield-Gabrieli et al., 2009). Excessive activation in DMN regions was suggested to increase the risk of thought disorders in schizophrenia patients (Whitfield-Gabrieli et al., 2009). Another study on temporal lobe epilepsy found increased FC in the left DMN regions in patients with right hippocampal sclerosis, which was interpreted as a compensatory mechanism (Zanao et al., 2021). In a study by Tao et al. (2017) on amnestic mild cognitive impairment (aMCI), they reported that patients with mild to moderate aMCI exhibited increased DMN FC value. Similarly, Bonnelle et al. (2011) found a relationship between impaired cognitive control and increased DMN activation in patients with traumatic brain injury. These studies suggest that excessive DMN activation may indeed represent a form of neural compensation, though it is not always beneficial. Alternatively, this objective alteration of DMN overactivation at the brain network level may not always contribute positively to symptom improvement, disease severity reduction, or cognitive function enhancement. This aligns with our findings to some extent, where we observed hyperactivation both within the DMN and between networks, which may negatively impact cognitive function. This phenomenon can be observed across a range of diseases, suggesting that it may exist within a broader clinical framework.

Our findings may provide valuable insights for research on “prehabilitation.” Desmurget et al. (2007) suggested that the brain demonstrates superior functional recovery when damage progresses slowly rather than acutely. Duffau, in a study of adult glioma patients, proposed that brain circuit reorganization allows extensive resection of “critical” regions without causing permanent deficits (Duffau, 2014). Additionally, cases of tumor recurrence and repeated surgeries have shown functional reallocation over time within the same patient (Duffau, 2014). These findings suggest that slow-progressing lesions offer a “window of opportunity” for brain plasticity, enabling the transfer of functions from affected areas to other regions.

Preoperative interventions to facilitate this transfer process (prehabilitation) could help neurosurgeons achieve maximal lesion resection with minimal long-term neurological deficits. Poologaindran et al. (2022) demonstrated the safety and efficacy of targeted neuromodulation guided by neuroimaging in addressing motor and language impairments in postoperative brain tumor patients. Similarly, case reports by Sughrue et al. (2022) highlighted the feasibility and effectiveness of prehabilitation. Our results indicate that nodes within the DMN, such as ACC, could serve as potential prehabilitation targets. Modulating the activation of the DMN may enhance prehabilitation in pediatric patients with temporal lobe lesions, potentially leading to improved postoperative outcomes.

## 5 Conclusion

Our study found that preoperative cognitive impairments in children with temporal lobe space-occupying lesions primarily involve deficits in working memory and sustained attention. The alterations in cerebrum networks are mainly characterized by hyperactivation of the DMN, and this hyperactivation is correlated to some extent with the impairments in working memory and sustained attention. Our research advances the understanding of the impact of temporal lobe space-occupying lesions on cognitive function in children and the underlying brain network mechanisms. This offers a distinctive and clinically significant reference for the development and application of preoperative interventions and tailored prehabilitation strategies moving forward.

## 6 Limitations

This research has some limitations. The sample size, heterogeneity of the sample and study design restrict the interpretation of its results. The results of the correlational analysis appear to lack stability, as they do not withstand multiple comparison correction. However, they may still provide valuable insights for future research, warranting further in-depth investigation. Moreover, children’s lower self-control poses difficulties in reducing head movement during extended MRI scans, leading to potential data loss or unusable results. Therefore, further extensive studies are required to confirm these findings.

## Data Availability

The raw data supporting the conclusions of this article will be made available by the authors, without undue reservation.

## Footnotes

1
http://rfmri.org/dpabi

2
http://www.fli.ion.ucl.ac.uk/spm

3
https://neuro-jena.github.io/cat/
