# Supplementary material for: Cognitive and cortical network alterations in pediatric temporal lobe space-occupying lesions: an fMRI study
Source: Front Hum Neurosci. 2024 Dec 9;18:1509899. doi: 10.3389/fnhum.2024.1509899 (PMC11663916; doi:10.3389/fnhum.2024.1509899)
Supplement: Supplementary file 1 [file Supplementary_file_1.docx]

**Supplementary Material 1:**

**The Method of mapping AAL atlas to macroscopic networks**

Due to the advancements in current neuroimaging and analysis techniques, the human brain has been divided into numerous macroscopic networks. However, there exists some degree of variation in the spatial topology and naming conventions across different studies^1^. Particularly when categorizing brain regions of brain atlases into different macroscopic networks, the results from various studies often show inconsistencies.

Based on Yeo's 7-network model and in conjunction with previous research and anatomical locations, we segmented the AAL-116 brain atlas into nine networks without disrupting each ROI and ensuring no overlap between ROIs^2,3^.

We initially aligned each Region of Interest (ROI) from the AAL-116 brain template with the Yeo-7 network template. Subsequently, we examined the proportion of the seven components of the Yeo network within each ROI and categorized each ROI into the network with the highest overlapping proportion, adhering to the principle of “winner-takes-all”.

Subsequently, we made adjustments based on previous research and anatomical considerations. The primary focus of these adjustments involved reclassifying regions that were clearly misclassified and addressing areas that could not be categorized due to minimal overlap between the AAL and Yeo templates.

The following literature references were consulted during the adjustment phase following the initial step, and the table below presents the final number of ROIs for each network.

Furthermore, we have provided an Excel spreadsheet that presents the correspondence between each specific ROI and the nine networks for future reference by researchers (see Supplementary Material 2).

The determination of these anatomical locations was confirmed by two neurosurgeons with over five years of professional experience.

The Cerebellar Network (CereN) and the Basal Ganglia Network (BGN) were determined based on their anatomical locations. The CereN comprises 26 ROIs, while the BGN consists of 8 ROIs.

Based on the anatomical locations of the DMN within Yeo's 7-network model^2^ and the core nodes of the DMN provided by Uddin et al^1^., in conjunction with previous researches on the default network^4-6^, a total of 18 nodes were identified.

The core regions of the SMN are located within the motor and somatosensory cortices^1^. Combining prior research and the anatomical positions within the Yeo network, we have identified a total of 14 nodes within the SMN^2,7^.

The core region of the VN lies within the occipital lobe^1^. Combining the researches conducted by Smith et al^8^. and Weiner et al^9^., along with the anatomical positions within the Yeo network for the VN, we have identified a total of 14 nodes.

In conjunction with the study conducted by Jimenez et al^10^., we have identified six nodes within the VAN.

Drawing upon the studies conducted by Uddin et al^1^., Vossel et al^11^., and Jimenez et al^10^., we have identified four nodes within the DAN.

Combining Catani et al^12^.'s comprehensive review of the limbic system and the anatomical positions within Yeo's limbic network, we have identified 16 nodes within the salience network.

Drawing upon the research conducted by Oliver et al^5^., anatomical observations within the Yeo network, and insights provided by Uddin et al^1^., we have ultimately identified 10 nodes within the FPN.

Network name, abbreviation, and number of nodes

| Network name | Abbreviations | Number of Nodes |
| --- | --- | --- |
| Visual Network | VN | 14 |
| Sensorimotor Network | SMN | 14 |
| Dorsal Attention Network | DAN | 4 |
| Ventral Attention Network | VAN | 6 |
| Limbic Network | LN | 16 |
| Fronto-parietal Network | FPN | 10 |
| Default Mode Network | DMN | 18 |
| Basal Ganglia Network | BGN | 8 |
| Cerebellar Network | CereN | 26 |

**Reference**

1. Uddin LQ, Yeo BTT, Spreng RN. Towards a Universal Taxonomy of Macro-scale Functional Human Brain Networks. *Brain topography*. 2019;32(6):926-942. doi:10.1007/s10548-019-00744-6

2. Yeo BT, Krienen FM, Sepulcre J, et al. The organization of the human cerebral cortex estimated by intrinsic functional connectivity. *Journal of neurophysiology*. Sep 2011;106(3):1125-65. doi:10.1152/jn.00338.2011

3. Tzourio-Mazoyer N, Landeau B, Papathanassiou D, et al. Automated anatomical labeling of activations in SPM using a macroscopic anatomical parcellation of the MNI MRI single-subject brain. *Neuroimage*. Jan 2002;15(1):273-89. doi:10.1006/nimg.2001.0978

4. Raichle ME. The brain's default mode network. *Annu Rev Neurosci*. Jul 8 2015;38:433-47. doi:10.1146/annurev-neuro-071013-014030

5. Oliver I, Hlinka J, Kopal J, Davidsen J. Quantifying the Variability in Resting-State Networks. *Entropy*. 2019;21(9)doi:10.3390/e21090882

6. Fair DA, Cohen AL, Power JD, et al. Functional brain networks develop from a "local to distributed" organization. *PLoS computational biology*. May 2009;5(5):e1000381. doi:10.1371/journal.pcbi.1000381

7. Chenji S, Jha S, Lee D, et al. Investigating Default Mode and Sensorimotor Network Connectivity in Amyotrophic Lateral Sclerosis. *PLoS One*. 2016;11(6):e0157443. doi:10.1371/journal.pone.0157443

8. Smith SM, Fox PT, Miller KL, et al. Correspondence of the brain's functional architecture during activation and rest. *Proceedings of the National Academy of Sciences of the United States of America*. Aug 4 2009;106(31):13040-5. doi:10.1073/pnas.0905267106

9. Weiner KS, Zilles K. The anatomical and functional specialization of the fusiform gyrus. *Neuropsychologia*. Mar 2016;83:48-62. doi:10.1016/j.neuropsychologia.2015.06.033

10. Jimenez AM, Lee J, Wynn JK, et al. Abnormal Ventral and Dorsal Attention Network Activity during Single and Dual Target Detection in Schizophrenia. *Front Psychol*. 2016;7:323. doi:10.3389/fpsyg.2016.00323

11. Vossel S, Geng JJ, Fink GR. Dorsal and ventral attention systems: distinct neural circuits but collaborative roles. *Neuroscientist*. Apr 2014;20(2):150-9. doi:10.1177/1073858413494269

12. Catani M, Dell'acqua F, Thiebaut de Schotten M. A revised limbic system model for memory, emotion and behaviour. *Neuroscience and biobehavioral reviews*. Sep 2013;37(8):1724-37. doi:10.1016/j.neubiorev.2013.07.001
