# Supplementary material for: Cognitive and cortical network alterations in pediatric temporal lobe space-occupying lesions: an fMRI study
Source: Front Hum Neurosci. 2024 Dec 9;18:1509899. doi: 10.3389/fnhum.2024.1509899 (PMC11663916; doi:10.3389/fnhum.2024.1509899)
Supplement: Supplementary file 2 [file Supplementary_file_2.pdf]

Supplementary Material 2:

AAL order	AAL Label	Network name
1	'Precentral_L'	Sensorimotor Network
2	'Precentral_R'	Sensorimotor Network
3	'Frontal_Sup_L'	Dorsal Attention
4	'Frontal_Sup_R'	Dorsal Attention
5	'Frontal_Sup_Orb_L'	Limbic
6	'Frontal_Sup_Orb_R'	Limbic
7	'Frontal_Mid_L'	Frontoparietal Network
8	'Frontal_Mid_R'	Frontoparietal Network
9	'Frontal_Mid_Orb_L'	Frontoparietal Network
10	'Frontal_Mid_Orb_R'	Frontoparietal Network
11	'Frontal_Inf_Oper_L'	Frontoparietal Network
12	'Frontal_Inf_Oper_R'	Frontoparietal Network
13	'Frontal_Inf_Tri_L'	Frontoparietal Network
14	'Frontal_Inf_Tri_R'	Frontoparietal Network
15	'Frontal_Inf_Orb_L'	Default mode Network
16	'Frontal_Inf_Orb_R'	Default mode Network
17	'Rolandic_Oper_L'	Sensorimotor Network
18	'Rolandic_Oper_R'	Sensorimotor Network
19	'Supp_Motor_Area_L'	Sensorimotor Network
20	'Supp_Motor_Area_R'	Sensorimotor Network
21	'Olfactory_L'	Limbic
22	'Olfactory_R'	Limbic
23	'Frontal_Sup_Medial_L'	Default mode Network
24	'Frontal_Sup_Medial_R'	Default mode Network
25	'Frontal_Med_Orb_L'	Default mode Network
26	'Frontal_Med_Orb_R'	Default mode Network
27	'Rectus_L'	Limbic
28	'Rectus_R'	Limbic
29	'Insula_L'	Ventral Attention
30	'Insula_R'	Ventral Attention
31	'Cingulum_Ant_L'	Default mode Network
32	'Cingulum_Ant_R'	Default mode Network
33	'Cingulum_Mid_L'	Ventral Attention
34	'Cingulum_Mid_R'	Ventral Attention
35	'Cingulum_Post_L'	Default mode Network
36	'Cingulum_Post_R'	Default Mode Network
37	'Hippocampus_L'	Limbic
38	'Hippocampus_R'	Limbic
39	'ParaHippocampal_L'	Default Mode Network
40	'ParaHippocampal_R'	Default Mode Network
41	'Amygdala_L'	Limbic
42	'Amygdala_R'	Limbic
43	'Calcarine_L'	Visual Network
44	'Calcarine_R'	Visual Network
45	'Cuneus_L'	Visual Network
46	'Cuneus_R'	Visual Network
47	'Lingual_L'	Visual Network
48	'Lingual_R'	Visual Network
49	'Occipital_Sup_L'	Visual Network
50	'Occipital_Sup_R'	Visual Network
51	'Occipital_Mid_L'	Visual Network
52	'Occipital_Mid_R'	Visual Network
53	'Occipital_Inf_L'	Visual Network
54	'Occipital_Inf_R'	Visual Network
55	'Fusiform_L'	Visual Network
56	'Fusiform_R'	Visual Network
57	'Postcentral_L'	Sensorimotor Network
58	'Postcentral_R'	Sensorimotor Network

59	'Parietal_Sup_L'	Dorsal Attention
60	'Parietal_Sup_R'	Dorsal Attention
61	'Parietal_Inf_L'	Frontoparietal Network
62	'Parietal_Inf_R'	Frontoparietal Network
63	'SupraMarginal_L'	Ventral Attention
64	'SupraMarginal_R'	Ventral Attention
65	'Angular_L'	Default mode Network
66	'Angular_R'	Default mode Network
67	'Precuneus_L'	Default mode Network
68	'Precuneus_R'	Default mode Network
69	'Paracentral_Lobule_L'	Sensorimotor Network
70	'Paracentral_Lobule_R'	Sensorimotor Network
71	'Caudate_L'	basal gangila
72	'Caudate_R'	basal gangila
73	'Putamen_L'	basal gangila
74	'Putamen_R'	basal gangila
75	'Pallidum_L'	basal gangila
76	'Pallidum_R'	basal gangila
77	'Thalamus_L'	basal gangila
78	'Thalamus_R'	basal gangila
79	'Heschl_L'	Sensorimotor Network
80	'Heschl_R'	Sensorimotor Network
81	'Temporal_Sup_L'	Sensorimotor Network
82	'Temporal_Sup_R'	Sensorimotor Network
83	'Temporal_Pole_Sup_L'	Limbic
84	'Temporal_Pole_Sup_R'	Limbic
85	'Temporal_Mid_L'	Default mode Network
86	'Temporal_Mid_R'	Default mode Network
87	'Temporal_Pole_Mid_L'	Limbic
88	'Temporal_Pole_Mid_R'	Limbic
89	'Temporal_Inf_L'	Limbic
90	'Temporal_Inf_R'	Limbic
91	'Cerebelum_Crus1_L'	cerebellum
92	'Cerebelum_Crus1_R'	cerebellum
93	'Cerebelum_Crus2_L'	cerebellum
94	'Cerebelum_Crus2_R'	cerebellum
95	'Cerebelum_3_L'	cerebellum
96	'Cerebelum_3_R'	cerebellum
97	'Cerebelum_4_5_L'	cerebellum
98	'Cerebelum_4_5_R'	cerebellum
99	'Cerebelum_6_L'	cerebellum
100	'Cerebelum_6_R'	cerebellum
101	'Cerebelum_7b_L'	cerebellum
102	'Cerebelum_7b_R'	cerebellum
103	'Cerebelum_8_L'	cerebellum
104	'Cerebelum_8_R'	cerebellum
105	'Cerebelum_9_L'	cerebellum
106	'Cerebelum_9_R'	cerebellum
107	'Cerebelum_10_L'	cerebellum
108	'Cerebelum_10_R'	cerebellum
109	'Vermis_1_2'	cerebellum
110	'Vermis_3'	cerebellum
111	'Vermis_4_5'	cerebellum
112	'Vermis_6'	cerebellum
113	'Vermis_7'	cerebellum
114	'Vermis_8'	cerebellum
115	'Vermis_9'	cerebellum
116	'Vermis_10'	cerebellum
