# Supplementary material for: Cognitive and cortical network alterations in pediatric temporal lobe space-occupying lesions: an fMRI study
Source: Front Hum Neurosci. 2024 Dec 9;18:1509899. doi: 10.3389/fnhum.2024.1509899 (PMC11663916; doi:10.3389/fnhum.2024.1509899)
Supplement: Supplementary file 3 [file Supplementary_file_3.docx]

Supplementary Material 3: Histopathological diagnoses of enrolled pediatric patients

| Subjects’ ID | Histopathological diagnoses^#^ | WHO grade^$^ |
| --- | --- | --- |
| sub001 | Ganglioglioma | Ⅰ |
| sub002 | Dysembryoplastic Neuroepithelial Tumor | Ⅰ |
| sub003 | Ganglioglioma | Ⅰ |
| sub004 | Pilomyxoid Astrocytoma | Ⅱ |
| sub005 | Astrocytoma | Ⅰ |
| sub006 | Cavernous Hemangioma |  |
| sub007 | Ganglioglioma | Ⅰ |
| sub008 | Dermoid Cyst |  |
| sub009 | Ganglioglioma | Ⅰ |
| sub010 | Cavernous Hemangioma |  |
| sub011 | Ganglioglioma | Ⅰ |
| sub012 | Atypical Meningioma | Ⅰ-Ⅱ |
| sub013 | Ewing Sarcoma | Ⅳ |
| sub014 | Ganglioglioma | Ⅰ |
| sub015 | Neuronal and mixed neuronal-glial tumors | Ⅰ |

# The histopathological diagnoses listed in this table are all based on the reports from the pathology department of our hospital.

$ We utilized the 2016 edition of the pathological grading system.
