# Supplementary material for: Cognitive and cortical network alterations in pediatric temporal lobe space-occupying lesions: an fMRI study
Source: Front Hum Neurosci. 2024 Dec 9;18:1509899. doi: 10.3389/fnhum.2024.1509899 (PMC11663916; doi:10.3389/fnhum.2024.1509899)

**Supplementary Material 4：**

**Ⅰ. Detailed results of imaging data analysis corrected by GRF:**

**1.ALFF:**

Cluster 1 -> Cluster Size (Voxels): 266; Cluster Size (mm^3): 7182

Peak Index: 16 34 32

Peak Coordinate (X Y Z): 45 -27 21

Peak Intensity: 8.50559

Label Include:

AAL -> Peak Label at [18] Rolandic_Oper_R

[64] SupraMarginal_R, 21.0526% (56)

[62] Parietal_Inf_R, 16.9173% (45)

[58] Postcentral_R, 15.7895% (42)

[82] Temporal_Sup_R, 15.0376% (40)

[18] Rolandic_Oper_R, 12.0301% (32)

[60] Parietal_Sup_R, 9.02256% (24)

[86] Temporal_Mid_R, 8.27068% (22)

[66] Angular_R, 1.12782% (3)

[2] Precentral_R, 0.75188% (2)

Cluster 2 -> Cluster Size (Voxels): 257; Cluster Size (mm^3): 6939

Peak Index: 26 64 31

Peak Coordinate (X Y Z): 15 63 18

Peak Intensity: 9.90976

Label Include:

AAL -> Peak Label at [4] Frontal_Sup_R

[4] Frontal_Sup_R, 36.965% (95)

[24] Frontal_Sup_Medial_R, 29.1829% (75)

[32] Cingulum_Ant_R, 14.786% (38)

[34] Cingulum_Mid_R, 10.5058% (27)

[8] Frontal_Mid_R, 5.05837% (13)

[6] Frontal_Sup_Orb_R, 3.50195% (9)

Cluster 3 -> Cluster Size (Voxels): 241; Cluster Size (mm^3): 6507

Peak Index: 30 61 41

Peak Coordinate (X Y Z): 3 54 48

Peak Intensity: -9.11116

Label Include:

AAL -> Peak Label at [24] Frontal_Sup_Medial_R

[23] Frontal_Sup_Medial_L, 44.3983% (107)

[3] Frontal_Sup_L, 35.6846% (86)

[5] Frontal_Sup_Orb_L, 9.54357% (23)

[7] Frontal_Mid_L, 4.97925% (12)

[24] Frontal_Sup_Medial_R, 3.73444% (9)

[9] Frontal_Mid_Orb_L, 1.24481% (3)

[25] Frontal_Med_Orb_L, 0.414938% (1)

Cluster 4 -> Cluster Size (Voxels): 163; Cluster Size (mm^3): 4401

Peak Index: 15 44 22

Peak Coordinate (X Y Z): 48 3 -9

Peak Intensity: -7.72814

Label Include:

AAL -> Peak Label at [82] Temporal_Sup_R

[86] Temporal_Mid_R, 39.2638% (64)

[90] Temporal_Inf_R, 28.8344% (47)

[84] Temporal_Pole_Sup_R, 15.9509% (26)

[82] Temporal_Sup_R, 7.97546% (13)

[88] Temporal_Pole_Mid_R, 7.97546% (13)

Cluster 5 -> Cluster Size (Voxels): 108; Cluster Size (mm^3): 2916

Peak Index: 32 53 24

Peak Coordinate (X Y Z): -3 30 -3

Peak Intensity: 7.52127

Label Include:

AAL -> Peak Label at [31] Cingulum_Ant_L

[31] Cingulum_Ant_L, 49.0741% (53)

[32] Cingulum_Ant_R, 24.0741% (26)

[25] Frontal_Med_Orb_L, 14.8148% (16)

[21] Olfactory_L, 6.48148% (7)

[23] Frontal_Sup_Medial_L, 2.77778% (3)

[22] Olfactory_R, 0.925926% (1)

[26] Frontal_Med_Orb_R, 0.925926% (1)

[27] Rectus_L, 0.925926% (1)

Cluster 6 -> Cluster Size (Voxels): 86; Cluster Size (mm^3): 2322

Peak Index: 15 47 33

Peak Coordinate (X Y Z): 48 12 24

Peak Intensity: -6.36803

Label Include:

AAL -> Peak Label at [12] Frontal_Inf_Oper_R

[2] Precentral_R, 65.1163% (56)

[12] Frontal_Inf_Oper_R, 19.7674% (17)

[18] Rolandic_Oper_R, 10.4651% (9)

[14] Frontal_Inf_Tri_R, 2.32558% (2)

[58] Postcentral_R, 2.32558% (2)

Cluster 7 -> Cluster Size (Voxels): 62; Cluster Size (mm^3): 1674

Peak Index: 49 40 17

Peak Coordinate (X Y Z): -54 -9 -24

Peak Intensity: -9.08484

Label Include:

AAL -> Peak Label at [89] Temporal_Inf_L

[85] Temporal_Mid_L, 69.3548% (43)

[89] Temporal_Inf_L, 29.0323% (18)

[87] Temporal_Pole_Mid_L, 1.6129% (1)

Cluster 8 -> Cluster Size (Voxels): 60; Cluster Size (mm^3): 1620

Peak Index: 44 24 45

Peak Coordinate (X Y Z): -39 -57 60

Peak Intensity: 5.508

Label Include:

AAL -> Peak Label at [61] Parietal_Inf_L

[59] Parietal_Sup_L, 65% (39)

[61] Parietal_Inf_L, 35% (21)

Cluster 9 -> Cluster Size (Voxels): 59; Cluster Size (mm^3): 1593

Peak Index: 46 24 21

Peak Coordinate (X Y Z): -45 -57 -12

Peak Intensity: 5.57475

Label Include:

AAL -> Peak Label at [53] Occipital_Inf_L

[89] Temporal_Inf_L, 33.8983% (20)

[53] Occipital_Inf_L, 23.7288% (14)

[55] Fusiform_L, 20.339% (12)

[85] Temporal_Mid_L, 13.5593% (8)

[51] Occipital_Mid_L, 8.47458% (5)

Cluster 10 -> Cluster Size (Voxels): 56; Cluster Size (mm^3): 1512

Peak Index: 32 64 18

Peak Coordinate (X Y Z): -3 63 -21

Peak Intensity: -6.50405

Label Include:

AAL -> Peak Label at [27] Rectus_L

[27] Rectus_L, 35.7143% (20)

[28] Rectus_R, 25% (14)

[25] Frontal_Med_Orb_L, 21.4286% (12)

[26] Frontal_Med_Orb_R, 16.0714% (9)

[6] Frontal_Sup_Orb_R, 1.78571% (1)

Cluster 11 -> Cluster Size (Voxels): 48; Cluster Size (mm^3): 1296

Peak Index: 53 37 31

Peak Coordinate (X Y Z): -66 -18 18

Peak Intensity: 5.57447

Label Include:

AAL -> Peak Label at [57] Postcentral_L

[57] Postcentral_L, 39.5833% (19)

[81] Temporal_Sup_L, 27.0833% (13)

[63] SupraMarginal_L, 25% (12)

[17] Rolandic_Oper_L, 4.16667% (2)

[79] Heschl_L, 4.16667% (2)

Cluster 12 -> Cluster Size (Voxels): 45; Cluster Size (mm^3): 1215

Peak Index: 18 21 32

Peak Coordinate (X Y Z): 39 -66 21

Peak Intensity: 6.90907

Label Include:

AAL -> Peak Label at [86] Temporal_Mid_R

[86] Temporal_Mid_R, 86.6667% (39)

[52] Occipital_Mid_R, 13.3333% (6)

Cluster 13 -> Cluster Size (Voxels): 41; Cluster Size (mm^3): 1107

Peak Index: 18 54 23

Peak Coordinate (X Y Z): 39 33 -6

Peak Intensity: -6.15377

Label Include:

AAL -> Peak Label at [16] Frontal_Inf_Orb_R

[16] Frontal_Inf_Orb_R, 78.0488% (32)

[30] Insula_R, 19.5122% (8)

[14] Frontal_Inf_Tri_R, 2.43902% (1)

Cluster 14 -> Cluster Size (Voxels): 37; Cluster Size (mm^3): 999

Peak Index: 12 20 32

Peak Coordinate (X Y Z): 57 -69 21

Peak Intensity: -6.77826

Label Include:

AAL -> Peak Label at [86] Temporal_Mid_R

[52] Occipital_Mid_R, 43.2432% (16)

[86] Temporal_Mid_R, 32.4324% (12)

[66] Angular_R, 24.3243% (9)

Cluster 15 -> Cluster Size (Voxels): 29; Cluster Size (mm^3): 783

Peak Index: 11 38 34

Peak Coordinate (X Y Z): 60 -15 27

Peak Intensity: 5.07395

Label Include:

AAL -> Peak Label at [64] SupraMarginal_R

[58] Postcentral_R, 51.7241% (15)

[82] Temporal_Sup_R, 27.5862% (8)

[64] SupraMarginal_R, 20.6897% (6)

Cluster 16 -> Cluster Size (Voxels): 28; Cluster Size (mm^3): 756

Peak Index: 48 27 23

Peak Coordinate (X Y Z): -51 -48 -6

Peak Intensity: 5.75612

Label Include:

AAL -> Peak Label at [89] Temporal_Inf_L

[85] Temporal_Mid_L, 64.2857% (18)

[89] Temporal_Inf_L, 35.7143% (10)

Cluster 17 -> Cluster Size (Voxels): 24; Cluster Size (mm^3): 648

Peak Index: 23 52 18

Peak Coordinate (X Y Z): 24 27 -21

Peak Intensity: 6.56975

Label Include:

AAL -> Peak Label at [16] Frontal_Inf_Orb_R

[16] Frontal_Inf_Orb_R, 70.8333% (17)

[6] Frontal_Sup_Orb_R, 20.8333% (5)

[10] Frontal_Mid_Orb_R, 8.33333% (2)

Cluster 18 -> Cluster Size (Voxels): 22; Cluster Size (mm^3): 594

Peak Index: 20 13 21

Peak Coordinate (X Y Z): 33 -90 -12

Peak Intensity: 5.69396

Label Include:

AAL -> Peak Label at [54] Occipital_Inf_R

[54] Occipital_Inf_R, 90.9091% (20)

[48] Lingual_R, 9.09091% (2)

**2.fALFF:**

Cluster 1 -> Cluster Size (Voxels): 117; Cluster Size (mm^3): 3159

Peak Index: 33 56 22

Peak Coordinate (X Y Z): -6 39 -9

Peak Intensity: 6.97207

Label Include:

AAL -> Peak Label at [25] Frontal_Med_Orb_L

[31] Cingulum_Ant_L, 36.7521% (43)

[32] Cingulum_Ant_R, 35.8974% (42)

[25] Frontal_Med_Orb_L, 19.6581% (23)

[26] Frontal_Med_Orb_R, 6.83761% (8)

[21] Olfactory_L, 0.854701% (1)

Cluster 2 -> Cluster Size (Voxels): 113; Cluster Size (mm^3): 3051

Peak Index: 15 18 35

Peak Coordinate (X Y Z): 48 -75 30

Peak Intensity: -7.09725

Label Include:

AAL -> Peak Label at [52] Occipital_Mid_R

[52] Occipital_Mid_R, 46.0177% (52)

[86] Temporal_Mid_R, 24.7788% (28)

[66] Angular_R, 23.8938% (27)

[64] SupraMarginal_R, 3.53982% (4)

[62] Parietal_Inf_R, 1.76991% (2)

Cluster 3 -> Cluster Size (Voxels): 82; Cluster Size (mm^3): 2214

Peak Index: 23 60 41

Peak Coordinate (X Y Z): 24 51 48

Peak Intensity: -6.10999

Label Include:

AAL -> Peak Label at [4] Frontal_Sup_R

[4] Frontal_Sup_R, 71.9512% (59)

[8] Frontal_Mid_R, 20.7317% (17)

[24] Frontal_Sup_Medial_R, 7.31707% (6)

Cluster 4 -> Cluster Size (Voxels): 66; Cluster Size (mm^3): 1782

Peak Index: 40 65 32

Peak Coordinate (X Y Z): -27 66 21

Peak Intensity: -5.79523

Label Include:

AAL -> Peak Label at [3] Frontal_Sup_L

[3] Frontal_Sup_L, 74.2424% (49)

[7] Frontal_Mid_L, 13.6364% (9)

[23] Frontal_Sup_Medial_L, 12.1212% (8)

Cluster 5 -> Cluster Size (Voxels): 63; Cluster Size (mm^3): 1701

Peak Index: 43 26 45

Peak Coordinate (X Y Z): -36 -51 60

Peak Intensity: 5.58958

Label Include:

AAL -> Peak Label at [59] Parietal_Sup_L

[61] Parietal_Inf_L, 57.1429% (36)

[59] Parietal_Sup_L, 42.8571% (27)

Cluster 6 -> Cluster Size (Voxels): 52; Cluster Size (mm^3): 1404

Peak Index: 27 62 32

Peak Coordinate (X Y Z): 12 57 21

Peak Intensity: 6.46538

Label Include:

AAL -> Peak Label at [24] Frontal_Sup_Medial_R

[24] Frontal_Sup_Medial_R, 61.5385% (32)

[4] Frontal_Sup_R, 38.4615% (20)

Cluster 7 -> Cluster Size (Voxels): 39; Cluster Size (mm^3): 1053

Peak Index: 29 65 19

Peak Coordinate (X Y Z): 6 66 -18

Peak Intensity: -6.96372

Label Include:

AAL -> Peak Label at [6] Frontal_Sup_Orb_R

[25] Frontal_Med_Orb_L, 38.4615% (15)

[27] Rectus_L, 35.8974% (14)

[28] Rectus_R, 12.8205% (5)

[6] Frontal_Sup_Orb_R, 7.69231% (3)

[26] Frontal_Med_Orb_R, 5.12821% (2)

Cluster 8 -> Cluster Size (Voxels): 34; Cluster Size (mm^3): 918

Peak Index: 27 22 37

Peak Coordinate (X Y Z): 12 -63 36

Peak Intensity: 6.30405

Label Include:

AAL -> Peak Label at [68] Precuneus_R

[68] Precuneus_R, 91.1765% (31)

[46] Cuneus_R, 8.82353% (3)

Cluster 9 -> Cluster Size (Voxels): 34; Cluster Size (mm^3): 918

Peak Index: 17 44 35

Peak Coordinate (X Y Z): 42 3 30

Peak Intensity: 6.93874

Label Include:

AAL -> Peak Label at [2] Precentral_R

[2] Precentral_R, 88.2353% (30)

[12] Frontal_Inf_Oper_R, 8.82353% (3)

[8] Frontal_Mid_R, 2.94118% (1)

Cluster 10 -> Cluster Size (Voxels): 30; Cluster Size (mm^3): 810

Peak Index: 36 64 28

Peak Coordinate (X Y Z): -15 63 9

Peak Intensity: 6.40088

Label Include:

AAL -> Peak Label at [3] Frontal_Sup_L

[3] Frontal_Sup_L, 50% (15)

[23] Frontal_Sup_Medial_L, 50% (15)

Cluster 11 -> Cluster Size (Voxels): 24; Cluster Size (mm^3): 648

Peak Index: 46 21 24

Peak Coordinate (X Y Z): -45 -66 -3

Peak Intensity: 5.7403

Label Include:

AAL -> Peak Label at [53] Occipital_Inf_L

[53] Occipital_Inf_L, 33.3333% (8)

[51] Occipital_Mid_L, 29.1667% (7)

[85] Temporal_Mid_L, 25% (6)

[89] Temporal_Inf_L, 12.5% (3)

Cluster 12 -> Cluster Size (Voxels): 23; Cluster Size (mm^3): 621

Peak Index: 21 23 39

Peak Coordinate (X Y Z): 30 -60 42

Peak Intensity: 6.11833

Label Include:

AAL -> Peak Label at [66] Angular_R

[66] Angular_R, 60.8696% (14)

[50] Occipital_Sup_R, 21.7391% (5)

[52] Occipital_Mid_R, 17.3913% (4)

**3.Reho:**

Cluster 1 -> Cluster Size (Voxels): 949; Cluster Size (mm^3): 25623

Peak Index: 32 67 32

Peak Coordinate (X Y Z): -3 72 21

Peak Intensity: -10.1281

Label Include:

AAL -> Peak Label at [23] Frontal_Sup_Medial_L

[4] Frontal_Sup_R, 24.9737% (237)

[23] Frontal_Sup_Medial_L, 19.5996% (186)

[24] Frontal_Sup_Medial_R, 17.3867% (165)

[8] Frontal_Mid_R, 14.2255% (135)

[3] Frontal_Sup_L, 13.4879% (128)

[25] Frontal_Med_Orb_L, 3.58272% (34)

[6] Frontal_Sup_Orb_R, 2.52898% (24)

[7] Frontal_Mid_L, 2.21286% (21)

[27] Rectus_L, 0.737619% (7)

[14] Frontal_Inf_Tri_R, 0.421496% (4)

[26] Frontal_Med_Orb_R, 0.421496% (4)

[10] Frontal_Mid_Orb_R, 0.210748% (2)

[28] Rectus_R, 0.210748% (2)

Cluster 2 -> Cluster Size (Voxels): 498; Cluster Size (mm^3): 13446

Peak Index: 18 16 39

Peak Coordinate (X Y Z): 39 -81 42

Peak Intensity: -7.89485

Label Include:

AAL -> Peak Label at [52] Occipital_Mid_R

[52] Occipital_Mid_R, 30.1205% (150)

[66] Angular_R, 23.2932% (116)

[50] Occipital_Sup_R, 14.0562% (70)

[60] Parietal_Sup_R, 11.0442% (55)

[86] Temporal_Mid_R, 10.8434% (54)

[46] Cuneus_R, 5.62249% (28)

[54] Occipital_Inf_R, 1.60643% (8)

[64] SupraMarginal_R, 1.60643% (8)

[62] Parietal_Inf_R, 1.00402% (5)

[68] Precuneus_R, 0.803213% (4)

Cluster 3 -> Cluster Size (Voxels): 148; Cluster Size (mm^3): 3996

Peak Index: 33 55 26

Peak Coordinate (X Y Z): -6 36 3

Peak Intensity: 12.1921

Label Include:

AAL -> Peak Label at [31] Cingulum_Ant_L

[31] Cingulum_Ant_L, 51.3514% (76)

[32] Cingulum_Ant_R, 22.973% (34)

[25] Frontal_Med_Orb_L, 18.2432% (27)

[26] Frontal_Med_Orb_R, 4.72973% (7)

[21] Olfactory_L, 1.35135% (2)

[27] Rectus_L, 1.35135% (2)

Cluster 4 -> Cluster Size (Voxels): 103; Cluster Size (mm^3): 2781

Peak Index: 39 15 40

Peak Coordinate (X Y Z): -24 -84 45

Peak Intensity: -6.17606

Label Include:

AAL -> Peak Label at [49] Occipital_Sup_L

[51] Occipital_Mid_L, 49.5146% (51)

[49] Occipital_Sup_L, 32.0388% (33)

[59] Parietal_Sup_L, 13.5922% (14)

[67] Precuneus_L, 2.91262% (3)

[45] Cuneus_L, 1.94175% (2)

Cluster 5 -> Cluster Size (Voxels): 77; Cluster Size (mm^3): 2079

Peak Index: 43 26 44

Peak Coordinate (X Y Z): -36 -51 57

Peak Intensity: 7.60338

Label Include:

AAL -> Peak Label at [61] Parietal_Inf_L

[61] Parietal_Inf_L, 54.5455% (42)

[59] Parietal_Sup_L, 45.4545% (35)

Cluster 6 -> Cluster Size (Voxels): 75; Cluster Size (mm^3): 2025

Peak Index: 18 44 37

Peak Coordinate (X Y Z): 39 3 36

Peak Intensity: 7.78672

Label Include:

AAL -> Peak Label at [2] Precentral_R

[2] Precentral_R, 70.6667% (53)

[18] Rolandic_Oper_R, 12% (9)

[12] Frontal_Inf_Oper_R, 10.6667% (8)

[8] Frontal_Mid_R, 6.66667% (5)

Cluster 7 -> Cluster Size (Voxels): 67; Cluster Size (mm^3): 1809

Peak Index: 16 20 24

Peak Coordinate (X Y Z): 45 -69 -3

Peak Intensity: 5.70806

Label Include:

AAL -> Peak Label at [90] Temporal_Inf_R

[52] Occipital_Mid_R, 40.2985% (27)

[86] Temporal_Mid_R, 38.806% (26)

[90] Temporal_Inf_R, 17.9104% (12)

[54] Occipital_Inf_R, 2.98507% (2)

Cluster 8 -> Cluster Size (Voxels): 61; Cluster Size (mm^3): 1647

Peak Index: 11 22 23

Peak Coordinate (X Y Z): 60 -63 -6

Peak Intensity: -6.10767

Label Include:

AAL -> Peak Label at [90] Temporal_Inf_R

[86] Temporal_Mid_R, 68.8525% (42)

[90] Temporal_Inf_R, 24.5902% (15)

[54] Occipital_Inf_R, 6.55738% (4)

Cluster 9 -> Cluster Size (Voxels): 58; Cluster Size (mm^3): 1566

Peak Index: 35 55 41

Peak Coordinate (X Y Z): -12 36 48

Peak Intensity: 7.16363

Label Include:

AAL -> Peak Label at [3] Frontal_Sup_L

[3] Frontal_Sup_L, 74.1379% (43)

[23] Frontal_Sup_Medial_L, 25.8621% (15)

Cluster 10 -> Cluster Size (Voxels): 56; Cluster Size (mm^3): 1512

Peak Index: 14 45 19

Peak Coordinate (X Y Z): 51 6 -18

Peak Intensity: 6.99736

Label Include:

AAL -> Peak Label at [88] Temporal_Pole_Mid_R

[84] Temporal_Pole_Sup_R, 41.0714% (23)

[86] Temporal_Mid_R, 26.7857% (15)

[88] Temporal_Pole_Mid_R, 25% (14)

[82] Temporal_Sup_R, 7.14286% (4)

Cluster 11 -> Cluster Size (Voxels): 54; Cluster Size (mm^3): 1458

Peak Index: 48 28 16

Peak Coordinate (X Y Z): -51 -45 -27

Peak Intensity: -7.2433

Label Include:

AAL -> Peak Label at [89] Temporal_Inf_L

[89] Temporal_Inf_L, 100% (54)

Cluster 12 -> Cluster Size (Voxels): 47; Cluster Size (mm^3): 1269

Peak Index: 38 61 23

Peak Coordinate (X Y Z): -21 54 -6

Peak Intensity: 5.63375

Label Include:

AAL -> Peak Label at [5] Frontal_Sup_Orb_L

[5] Frontal_Sup_Orb_L, 55.3191% (26)

[3] Frontal_Sup_L, 25.5319% (12)

[23] Frontal_Sup_Medial_L, 12.766% (6)

[9] Frontal_Mid_Orb_L, 4.25532% (2)

[7] Frontal_Mid_L, 2.12766% (1)

**4.SBA:**

**ROI1:**

Cluster 1 -> Cluster Size (Voxels): 123; Cluster Size (mm^3): 3321

Peak Index: 33 55 24

Peak Coordinate (X Y Z): -6 36 -3

Peak Intensity: 5.52011

Label Include:

AAL -> Peak Label at [31] Cingulum_Ant_L

[27] Rectus_L, 35.7724% (44)

[31] Cingulum_Ant_L, 26.0163% (32)

[28] Rectus_R, 13.0081% (16)

[25] Frontal_Med_Orb_L, 10.5691% (13)

[32] Cingulum_Ant_R, 9.7561% (12)

[26] Frontal_Med_Orb_R, 3.25203% (4)

[21] Olfactory_L, 1.62602% (2)

Cluster 2 -> Cluster Size (Voxels): 110; Cluster Size (mm^3): 2970

Peak Index: 30 47 47

Peak Coordinate (X Y Z): 3 12 66

Peak Intensity: 6.90566

Label Include:

AAL -> Peak Label at [20] Supp_Motor_Area_R

[20] Supp_Motor_Area_R, 53.6364% (59)

[19] Supp_Motor_Area_L, 30.9091% (34)

[4] Frontal_Sup_R, 8.18182% (9)

[23] Frontal_Sup_Medial_L, 6.36364% (7)

[3] Frontal_Sup_L, 0.909091% (1)

**ROI2:**

Cluster 1 -> Cluster Size (Voxels): 463; Cluster Size (mm^3): 12501

Peak Index: 34 55 24

Peak Coordinate (X Y Z): -9 36 -3

Peak Intensity: 9.24292

Label Include:

AAL -> Peak Label at [31] Cingulum_Ant_L

[31] Cingulum_Ant_L, 31.1015% (144)

[32] Cingulum_Ant_R, 13.8229% (64)

[23] Frontal_Sup_Medial_L, 11.879% (55)

[25] Frontal_Med_Orb_L, 9.50324% (44)

[4] Frontal_Sup_R, 6.69546% (31)

[24] Frontal_Sup_Medial_R, 6.47948% (30)

[3] Frontal_Sup_L, 5.61555% (26)

[26] Frontal_Med_Orb_R, 5.39957% (25)

[21] Olfactory_L, 3.02376% (14)

[27] Rectus_L, 3.02376% (14)

[8] Frontal_Mid_R, 2.59179% (12)

[28] Rectus_R, 0.647948% (3)

[22] Olfactory_R, 0.215983% (1)

Cluster 2 -> Cluster Size (Voxels): 198; Cluster Size (mm^3): 5346

Peak Index: 33 28 27

Peak Coordinate (X Y Z): -6 -45 6

Peak Intensity: 5.91381

Label Include:

AAL -> Peak Label at [43] Calcarine_L

[43] Calcarine_L, 22.7273% (45)

[67] Precuneus_L, 22.2222% (44)

[48] Lingual_R, 17.1717% (34)

[68] Precuneus_R, 15.1515% (30)

[44] Calcarine_R, 10.6061% (21)

[47] Lingual_L, 5.55556% (11)

[45] Cuneus_L, 2.52525% (5)

[35] Cingulum_Post_L, 2.0202% (4)

[36] Cingulum_Post_R, 2.0202% (4)

Cluster 3 -> Cluster Size (Voxels): 174; Cluster Size (mm^3): 4698

Peak Index: 35 54 42

Peak Coordinate (X Y Z): -12 33 51

Peak Intensity: 6.30986

Label Include:

AAL -> Peak Label at [3] Frontal_Sup_L

[3] Frontal_Sup_L, 62.069% (108)

[7] Frontal_Mid_L, 28.7356% (50)

[23] Frontal_Sup_Medial_L, 8.62069% (15)

[19] Supp_Motor_Area_L, 0.574713% (1)

Cluster 4 -> Cluster Size (Voxels): 151; Cluster Size (mm^3): 4077

Peak Index: 31 25 46

Peak Coordinate (X Y Z): 0 -54 63

Peak Intensity: 5.7037

Label Include:

AAL -> Peak Label at [67] Precuneus_L

[33] Cingulum_Mid_L, 39.7351% (60)

[67] Precuneus_L, 27.1523% (41)

[68] Precuneus_R, 13.245% (20)

[35] Cingulum_Post_L, 10.596% (16)

[69] Paracentral_Lobule_L, 5.96026% (9)

[34] Cingulum_Mid_R, 2.64901% (4)

[70] Paracentral_Lobule_R, 0.662252% (1)

Cluster 5 -> Cluster Size (Voxels): 109; Cluster Size (mm^3): 2943

Peak Index: 47 22 32

Peak Coordinate (X Y Z): -48 -63 21

Peak Intensity: 5.40615

Label Include:

AAL -> Peak Label at [85] Temporal_Mid_L

[85] Temporal_Mid_L, 48.6239% (53)

[65] Angular_L, 33.0275% (36)

[51] Occipital_Mid_L, 18.3486% (20)

Cluster 6 -> Cluster Size (Voxels): 78; Cluster Size (mm^3): 2106

Peak Index: 15 25 32

Peak Coordinate (X Y Z): 48 -54 21

Peak Intensity: 5.15548

Label Include:

AAL -> Peak Label at [86] Temporal_Mid_R

[66] Angular_R, 48.7179% (38)

[86] Temporal_Mid_R, 34.6154% (27)

[82] Temporal_Sup_R, 15.3846% (12)

[62] Parietal_Inf_R, 1.28205% (1)

Cluster 7 -> Cluster Size (Voxels): 65; Cluster Size (mm^3): 1755

Peak Index: 46 34 30

Peak Coordinate (X Y Z): -45 -27 15

Peak Intensity: 4.33144

Label Include:

AAL -> Peak Label at [17] Rolandic_Oper_L

[81] Temporal_Sup_L, 84.6154% (55)

[85] Temporal_Mid_L, 10.7692% (7)

[17] Rolandic_Oper_L, 4.61538% (3)

**ROI4:**

Cluster 1 -> Cluster Size (Voxels): 159; Cluster Size (mm^3): 4293

Peak Index: 47 54 37

Peak Coordinate (X Y Z): -48 33 36

Peak Intensity: -8.63717

Label Include:

AAL -> Peak Label at [7] Frontal_Mid_L

[7] Frontal_Mid_L, 75.4717% (120)

[13] Frontal_Inf_Tri_L, 18.8679% (30)

[3] Frontal_Sup_L, 5.03145% (8)

[11] Frontal_Inf_Oper_L, 0.628931% (1)

Cluster 2 -> Cluster Size (Voxels): 156; Cluster Size (mm^3): 4212

Peak Index: 26 24 38

Peak Coordinate (X Y Z): 15 -57 39

Peak Intensity: -6.18592

Label Include:

AAL -> Peak Label at [68] Precuneus_R

[67] Precuneus_L, 48.7179% (76)

[68] Precuneus_R, 48.7179% (76)

[46] Cuneus_R, 1.92308% (3)

[45] Cuneus_L, 0.641026% (1)

Cluster 3 -> Cluster Size (Voxels): 89; Cluster Size (mm^3): 2403

Peak Index: 47 30 38

Peak Coordinate (X Y Z): -48 -39 39

Peak Intensity: -5.49465

Label Include:

AAL -> Peak Label at [61] Parietal_Inf_L

[61] Parietal_Inf_L, 93.2584% (83)

[65] Angular_L, 5.61798% (5)

[63] SupraMarginal_L, 1.1236% (1)

Cluster 4 -> Cluster Size (Voxels): 82; Cluster Size (mm^3): 2214

Peak Index: 18 55 40

Peak Coordinate (X Y Z): 39 36 45

Peak Intensity: -6.91476

Label Include:

AAL -> Peak Label at [8] Frontal_Mid_R

[8] Frontal_Mid_R, 91.4634% (75)

[4] Frontal_Sup_R, 8.53659% (7)

Cluster 5 -> Cluster Size (Voxels): 50; Cluster Size (mm^3): 1350

Peak Index: 46 16 33

Peak Coordinate (X Y Z): -45 -81 24

Peak Intensity: -6.08406

Label Include:

AAL -> Peak Label at [51] Occipital_Mid_L

[51] Occipital_Mid_L, 94% (47)

[85] Temporal_Mid_L, 6% (3)

Cluster 6 -> Cluster Size (Voxels): 49; Cluster Size (mm^3): 1323

Peak Index: 21 20 39

Peak Coordinate (X Y Z): 30 -69 42

Peak Intensity: -4.66582

Label Include:

AAL -> Peak Label at [50] Occipital_Sup_R

[50] Occipital_Sup_R, 38.7755% (19)

[60] Parietal_Sup_R, 30.6122% (15)

[66] Angular_R, 24.4898% (12)

[52] Occipital_Mid_R, 6.12245% (3)

**Ⅱ. Detailed results of imaging data analysis corrected by FDR:**

**1.ALFF:**

Cluster 1 -> Cluster Size (Voxels): 953; Cluster Size (mm^3): 25731

Peak Index: 16 34 32

Peak Coordinate (X Y Z): 45 -27 21

Peak Intensity: 8.50559

Label Include:

AAL -> Peak Label at [18] Rolandic_Oper_R

[58] Postcentral_R, 16.6842% (159)

[64] SupraMarginal_R, 14.9003% (142)

[86] Temporal_Mid_R, 11.7524% (112)

[60] Parietal_Sup_R, 11.0178% (105)

[62] Parietal_Inf_R, 10.0735% (96)

[82] Temporal_Sup_R, 10.0735% (96)

[18] Rolandic_Oper_R, 7.66002% (73)

[66] Angular_R, 7.03043% (67)

[52] Occipital_Mid_R, 3.46275% (33)

[2] Precentral_R, 2.72823% (26)

[90] Temporal_Inf_R, 1.67891% (16)

[50] Occipital_Sup_R, 1.36411% (13)

[30] Insula_R, 1.04932% (10)

[54] Occipital_Inf_R, 0.419727% (4)

[84] Temporal_Pole_Sup_R, 0.104932% (1)

Cluster 2 -> Cluster Size (Voxels): 505; Cluster Size (mm^3): 13635

Peak Index: 30 61 41

Peak Coordinate (X Y Z): 3 54 48

Peak Intensity: -9.11116

Label Include:

AAL -> Peak Label at [24] Frontal_Sup_Medial_R

[23] Frontal_Sup_Medial_L, 33.4653% (169)

[3] Frontal_Sup_L, 24.7525% (125)

[4] Frontal_Sup_R, 6.33663% (32)

[5] Frontal_Sup_Orb_L, 6.13861% (31)

[27] Rectus_L, 5.74257% (29)

[25] Frontal_Med_Orb_L, 4.55446% (23)

[7] Frontal_Mid_L, 4.35644% (22)

[26] Frontal_Med_Orb_R, 4.15842% (21)

[24] Frontal_Sup_Medial_R, 3.76238% (19)

[28] Rectus_R, 3.56436% (18)

[8] Frontal_Mid_R, 1.58416% (8)

[6] Frontal_Sup_Orb_R, 0.792079% (4)

[9] Frontal_Mid_Orb_L, 0.792079% (4)

Cluster 3 -> Cluster Size (Voxels): 481; Cluster Size (mm^3): 12987

Peak Index: 26 64 31

Peak Coordinate (X Y Z): 15 63 18

Peak Intensity: 9.90976

Label Include:

AAL -> Peak Label at [4] Frontal_Sup_R

[4] Frontal_Sup_R, 27.6507% (133)

[24] Frontal_Sup_Medial_R, 25.7796% (124)

[34] Cingulum_Mid_R, 15.3846% (74)

[32] Cingulum_Ant_R, 13.5135% (65)

[20] Supp_Motor_Area_R, 6.02911% (29)

[6] Frontal_Sup_Orb_R, 5.61331% (27)

[8] Frontal_Mid_R, 4.5738% (22)

[23] Frontal_Sup_Medial_L, 0.623701% (3)

[19] Supp_Motor_Area_L, 0.4158% (2)

[26] Frontal_Med_Orb_R, 0.4158% (2)

Cluster 4 -> Cluster Size (Voxels): 411; Cluster Size (mm^3): 11097

Peak Index: 48 27 23

Peak Coordinate (X Y Z): -51 -48 -6

Peak Intensity: 5.75612

Label Include:

AAL -> Peak Label at [89] Temporal_Inf_L

[85] Temporal_Mid_L, 29.1971% (120)

[89] Temporal_Inf_L, 20.9246% (86)

[57] Postcentral_L, 10.7056% (44)

[81] Temporal_Sup_L, 10.219% (42)

[53] Occipital_Inf_L, 8.51582% (35)

[55] Fusiform_L, 8.51582% (35)

[63] SupraMarginal_L, 6.81265% (28)

[51] Occipital_Mid_L, 3.16302% (13)

[17] Rolandic_Oper_L, 0.973236% (4)

[79] Heschl_L, 0.973236% (4)

Cluster 5 -> Cluster Size (Voxels): 323; Cluster Size (mm^3): 8721

Peak Index: 15 44 22

Peak Coordinate (X Y Z): 48 3 -9

Peak Intensity: -7.72814

Label Include:

AAL -> Peak Label at [82] Temporal_Sup_R

[86] Temporal_Mid_R, 28.483% (92)

[90] Temporal_Inf_R, 22.6006% (73)

[84] Temporal_Pole_Sup_R, 16.7183% (54)

[88] Temporal_Pole_Mid_R, 13.9319% (45)

[30] Insula_R, 11.1455% (36)

[82] Temporal_Sup_R, 6.19195% (20)

[56] Fusiform_R, 0.928793% (3)

Cluster 6 -> Cluster Size (Voxels): 224; Cluster Size (mm^3): 6048

Peak Index: 15 47 33

Peak Coordinate (X Y Z): 48 12 24

Peak Intensity: -6.36803

Label Include:

AAL -> Peak Label at [12] Frontal_Inf_Oper_R

[2] Precentral_R, 41.0714% (92)

[12] Frontal_Inf_Oper_R, 19.6429% (44)

[18] Rolandic_Oper_R, 11.1607% (25)

[14] Frontal_Inf_Tri_R, 8.03571% (18)

[8] Frontal_Mid_R, 7.14286% (16)

[4] Frontal_Sup_R, 6.69643% (15)

[58] Postcentral_R, 5.80357% (13)

[20] Supp_Motor_Area_R, 0.446429% (1)

Cluster 7 -> Cluster Size (Voxels): 217; Cluster Size (mm^3): 5859

Peak Index: 32 53 24

Peak Coordinate (X Y Z): -3 30 -3

Peak Intensity: 7.52127

Label Include:

AAL -> Peak Label at [31] Cingulum_Ant_L

[31] Cingulum_Ant_L, 40.553% (88)

[32] Cingulum_Ant_R, 22.5806% (49)

[25] Frontal_Med_Orb_L, 12.4424% (27)

[23] Frontal_Sup_Medial_L, 8.75576% (19)

[21] Olfactory_L, 8.29493% (18)

[22] Olfactory_R, 3.22581% (7)

[26] Frontal_Med_Orb_R, 1.84332% (4)

[27] Rectus_L, 1.38249% (3)

[24] Frontal_Sup_Medial_R, 0.921659% (2)

Cluster 8 -> Cluster Size (Voxels): 203; Cluster Size (mm^3): 5481

Peak Index: 44 24 45

Peak Coordinate (X Y Z): -39 -57 60

Peak Intensity: 5.508

Label Include:

AAL -> Peak Label at [61] Parietal_Inf_L

[59] Parietal_Sup_L, 38.4236% (78)

[61] Parietal_Inf_L, 37.4384% (76)

[57] Postcentral_L, 23.6453% (48)

[63] SupraMarginal_L, 0.492611% (1)

Cluster 9 -> Cluster Size (Voxels): 101; Cluster Size (mm^3): 2727

Peak Index: 49 40 17

Peak Coordinate (X Y Z): -54 -9 -24

Peak Intensity: -9.08484

Label Include:

AAL -> Peak Label at [89] Temporal_Inf_L

[85] Temporal_Mid_L, 57.4257% (58)

[89] Temporal_Inf_L, 40.5941% (41)

[87] Temporal_Pole_Mid_L, 1.9802% (2)

Cluster 10 -> Cluster Size (Voxels): 98; Cluster Size (mm^3): 2646

Peak Index: 12 20 32

Peak Coordinate (X Y Z): 57 -69 21

Peak Intensity: -6.77826

Label Include:

AAL -> Peak Label at [86] Temporal_Mid_R

[52] Occipital_Mid_R, 30.6122% (30)

[50] Occipital_Sup_R, 23.4694% (23)

[66] Angular_R, 20.4082% (20)

[86] Temporal_Mid_R, 19.3878% (19)

[46] Cuneus_R, 6.12245% (6)

Cluster 11 -> Cluster Size (Voxels): 95; Cluster Size (mm^3): 2565

Peak Index: 18 54 23

Peak Coordinate (X Y Z): 39 33 -6

Peak Intensity: -6.15377

Label Include:

AAL -> Peak Label at [16] Frontal_Inf_Orb_R

[16] Frontal_Inf_Orb_R, 52.6316% (50)

[30] Insula_R, 31.5789% (30)

[12] Frontal_Inf_Oper_R, 7.36842% (7)

[14] Frontal_Inf_Tri_R, 5.26316% (5)

[18] Rolandic_Oper_R, 3.15789% (3)

Cluster 12 -> Cluster Size (Voxels): 89; Cluster Size (mm^3): 2403

Peak Index: 33 54 33

Peak Coordinate (X Y Z): -6 33 24

Peak Intensity: -6.70044

Label Include:

AAL -> Peak Label at [31] Cingulum_Ant_L

[33] Cingulum_Mid_L, 31.4607% (28)

[19] Supp_Motor_Area_L, 28.0899% (25)

[31] Cingulum_Ant_L, 22.4719% (20)

[23] Frontal_Sup_Medial_L, 15.7303% (14)

[3] Frontal_Sup_L, 2.24719% (2)

Cluster 13 -> Cluster Size (Voxels): 87; Cluster Size (mm^3): 2349

Peak Index: 26 26 37

Peak Coordinate (X Y Z): 15 -51 36

Peak Intensity: -4.88286

Label Include:

AAL -> Peak Label at [68] Precuneus_R

[34] Cingulum_Mid_R, 65.5172% (57)

[68] Precuneus_R, 34.4828% (30)

Cluster 14 -> Cluster Size (Voxels): 70; Cluster Size (mm^3): 1890

Peak Index: 36 33 39

Peak Coordinate (X Y Z): -15 -30 42

Peak Intensity: -4.19584

Label Include:

AAL -> Peak Label at [33] Cingulum_Mid_L

[33] Cingulum_Mid_L, 92.8571% (65)

[19] Supp_Motor_Area_L, 5.71429% (4)

[35] Cingulum_Post_L, 1.42857% (1)

Cluster 15 -> Cluster Size (Voxels): 53; Cluster Size (mm^3): 1431

Peak Index: 50 45 30

Peak Coordinate (X Y Z): -57 6 15

Peak Intensity: 4.52446

Label Include:

AAL -> Peak Label at [1] Precentral_L

[1] Precentral_L, 64.1509% (34)

[11] Frontal_Inf_Oper_L, 15.0943% (8)

[17] Rolandic_Oper_L, 15.0943% (8)

[57] Postcentral_L, 5.66038% (3)

Cluster 16 -> Cluster Size (Voxels): 51; Cluster Size (mm^3): 1377

Peak Index: 23 52 18

Peak Coordinate (X Y Z): 24 27 -21

Peak Intensity: 6.56975

Label Include:

AAL -> Peak Label at [16] Frontal_Inf_Orb_R

[16] Frontal_Inf_Orb_R, 49.0196% (25)

[6] Frontal_Sup_Orb_R, 45.098% (23)

[10] Frontal_Mid_Orb_R, 5.88235% (3)

**2.fALFF:**

Cluster 1 -> Cluster Size (Voxels): 270; Cluster Size (mm^3): 7290

Peak Index: 23 60 41

Peak Coordinate (X Y Z): 24 51 48

Peak Intensity: -6.10999

Label Include:

AAL -> Peak Label at [4] Frontal_Sup_R

[4] Frontal_Sup_R, 28.1481% (76)

[3] Frontal_Sup_L, 26.2963% (71)

[23] Frontal_Sup_Medial_L, 13.7037% (37)

[24] Frontal_Sup_Medial_R, 11.8519% (32)

[7] Frontal_Mid_L, 10.7407% (29)

[8] Frontal_Mid_R, 9.25926% (25)

Cluster 2 -> Cluster Size (Voxels): 174; Cluster Size (mm^3): 4698

Peak Index: 15 18 35

Peak Coordinate (X Y Z): 48 -75 30

Peak Intensity: -7.09725

Label Include:

AAL -> Peak Label at [52] Occipital_Mid_R

[52] Occipital_Mid_R, 39.0805% (68)

[66] Angular_R, 21.2644% (37)

[86] Temporal_Mid_R, 19.5402% (34)

[50] Occipital_Sup_R, 9.1954% (16)

[46] Cuneus_R, 6.89655% (12)

[64] SupraMarginal_R, 2.87356% (5)

[62] Parietal_Inf_R, 1.14943% (2)

Cluster 3 -> Cluster Size (Voxels): 162; Cluster Size (mm^3): 4374

Peak Index: 33 56 22

Peak Coordinate (X Y Z): -6 39 -9

Peak Intensity: 6.97207

Label Include:

AAL -> Peak Label at [25] Frontal_Med_Orb_L

[32] Cingulum_Ant_R, 35.8025% (58)

[31] Cingulum_Ant_L, 35.1852% (57)

[25] Frontal_Med_Orb_L, 20.9877% (34)

[26] Frontal_Med_Orb_R, 6.79012% (11)

[21] Olfactory_L, 0.617284% (1)

[27] Rectus_L, 0.617284% (1)

Cluster 4 -> Cluster Size (Voxels): 93; Cluster Size (mm^3): 2511

Peak Index: 43 26 45

Peak Coordinate (X Y Z): -36 -51 60

Peak Intensity: 5.58958

Label Include:

AAL -> Peak Label at [59] Parietal_Sup_L

[61] Parietal_Inf_L, 58.0645% (54)

[59] Parietal_Sup_L, 41.9355% (39)

Cluster 5 -> Cluster Size (Voxels): 65; Cluster Size (mm^3): 1755

Peak Index: 27 62 32

Peak Coordinate (X Y Z): 12 57 21

Peak Intensity: 6.46538

Label Include:

AAL -> Peak Label at [24] Frontal_Sup_Medial_R

[24] Frontal_Sup_Medial_R, 61.5385% (40)

[4] Frontal_Sup_R, 38.4615% (25)

Cluster 6 -> Cluster Size (Voxels): 52; Cluster Size (mm^3): 1404

Peak Index: 29 65 19

Peak Coordinate (X Y Z): 6 66 -18

Peak Intensity: -6.96372

Label Include:

AAL -> Peak Label at [6] Frontal_Sup_Orb_R

[25] Frontal_Med_Orb_L, 36.5385% (19)

[27] Rectus_L, 32.6923% (17)

[28] Rectus_R, 15.3846% (8)

[6] Frontal_Sup_Orb_R, 11.5385% (6)

[26] Frontal_Med_Orb_R, 3.84615% (2)

Cluster 7 -> Cluster Size (Voxels): 51; Cluster Size (mm^3): 1377

Peak Index: 21 23 39

Peak Coordinate (X Y Z): 30 -60 42

Peak Intensity: 6.11833

Label Include:

AAL -> Peak Label at [66] Angular_R

[66] Angular_R, 35.2941% (18)

[60] Parietal_Sup_R, 19.6078% (10)

[68] Precuneus_R, 19.6078% (10)

[50] Occipital_Sup_R, 17.6471% (9)

[52] Occipital_Mid_R, 7.84314% (4)

**3.Reho:**

Cluster 1 -> Cluster Size (Voxels): 1677; Cluster Size (mm^3): 45279

Peak Index: 32 67 32

Peak Coordinate (X Y Z): -3 72 21

Peak Intensity: -10.1281

Label Include:

AAL -> Peak Label at [23] Frontal_Sup_Medial_L

[4] Frontal_Sup_R, 21.6458% (363)

[8] Frontal_Mid_R, 18.6047% (312)

[23] Frontal_Sup_Medial_L, 14.1324% (237)

[24] Frontal_Sup_Medial_R, 13.9535% (234)

[3] Frontal_Sup_L, 10.3757% (174)

[7] Frontal_Mid_L, 3.81634% (64)

[25] Frontal_Med_Orb_L, 3.27967% (55)

[14] Frontal_Inf_Tri_R, 3.22004% (54)

[32] Cingulum_Ant_R, 3.04114% (51)

[6] Frontal_Sup_Orb_R, 2.62373% (44)

[26] Frontal_Med_Orb_R, 1.72928% (29)

[10] Frontal_Mid_Orb_R, 1.49076% (25)

[27] Rectus_L, 0.834824% (14)

[28] Rectus_R, 0.596303% (10)

[20] Supp_Motor_Area_R, 0.357782% (6)

[34] Cingulum_Mid_R, 0.298151% (5)

Cluster 2 -> Cluster Size (Voxels): 774; Cluster Size (mm^3): 20898

Peak Index: 18 16 39

Peak Coordinate (X Y Z): 39 -81 42

Peak Intensity: -7.89485

Label Include:

AAL -> Peak Label at [52] Occipital_Mid_R

[52] Occipital_Mid_R, 23.7726% (184)

[86] Temporal_Mid_R, 18.863% (146)

[66] Angular_R, 18.6047% (144)

[50] Occipital_Sup_R, 13.1783% (102)

[60] Parietal_Sup_R, 9.04393% (70)

[46] Cuneus_R, 5.16796% (40)

[54] Occipital_Inf_R, 2.97158% (23)

[90] Temporal_Inf_R, 2.71318% (21)

[64] SupraMarginal_R, 1.80879% (14)

[62] Parietal_Inf_R, 1.67959% (13)

[68] Precuneus_R, 1.29199% (10)

[82] Temporal_Sup_R, 0.904393% (7)

Cluster 3 -> Cluster Size (Voxels): 256; Cluster Size (mm^3): 6912

Peak Index: 33 55 26

Peak Coordinate (X Y Z): -6 36 3

Peak Intensity: 12.1921

Label Include:

AAL -> Peak Label at [31] Cingulum_Ant_L

[31] Cingulum_Ant_L, 37.8906% (97)

[32] Cingulum_Ant_R, 23.8281% (61)

[25] Frontal_Med_Orb_L, 14.8438% (38)

[27] Rectus_L, 11.7188% (30)

[26] Frontal_Med_Orb_R, 7.42188% (19)

[28] Rectus_R, 3.125% (8)

[21] Olfactory_L, 1.17188% (3)

Cluster 4 -> Cluster Size (Voxels): 228; Cluster Size (mm^3): 6156

Peak Index: 43 26 44

Peak Coordinate (X Y Z): -36 -51 57

Peak Intensity: 7.60338

Label Include:

AAL -> Peak Label at [61] Parietal_Inf_L

[61] Parietal_Inf_L, 42.5439% (97)

[59] Parietal_Sup_L, 33.7719% (77)

[65] Angular_L, 21.0526% (48)

[67] Precuneus_L, 2.19298% (5)

[51] Occipital_Mid_L, 0.438596% (1)

Cluster 5 -> Cluster Size (Voxels): 214; Cluster Size (mm^3): 5778

Peak Index: 35 55 41

Peak Coordinate (X Y Z): -12 36 48

Peak Intensity: 7.16363

Label Include:

AAL -> Peak Label at [3] Frontal_Sup_L

[3] Frontal_Sup_L, 46.2617% (99)

[23] Frontal_Sup_Medial_L, 21.028% (45)

[5] Frontal_Sup_Orb_L, 17.2897% (37)

[9] Frontal_Mid_Orb_L, 8.41121% (18)

[7] Frontal_Mid_L, 7.00935% (15)

Cluster 6 -> Cluster Size (Voxels): 189; Cluster Size (mm^3): 5103

Peak Index: 16 20 24

Peak Coordinate (X Y Z): 45 -69 -3

Peak Intensity: 5.70806

Label Include:

AAL -> Peak Label at [90] Temporal_Inf_R

[86] Temporal_Mid_R, 42.328% (80)

[52] Occipital_Mid_R, 30.1587% (57)

[54] Occipital_Inf_R, 10.582% (20)

[66] Angular_R, 7.93651% (15)

[90] Temporal_Inf_R, 7.93651% (15)

[56] Fusiform_R, 0.529101% (1)

[62] Parietal_Inf_R, 0.529101% (1)

Cluster 7 -> Cluster Size (Voxels): 178; Cluster Size (mm^3): 4806

Peak Index: 39 15 40

Peak Coordinate (X Y Z): -24 -84 45

Peak Intensity: -6.17606

Label Include:

AAL -> Peak Label at [49] Occipital_Sup_L

[51] Occipital_Mid_L, 46.0674% (82)

[49] Occipital_Sup_L, 25.8427% (46)

[59] Parietal_Sup_L, 11.7978% (21)

[67] Precuneus_L, 8.98876% (16)

[45] Cuneus_L, 5.05618% (9)

[85] Temporal_Mid_L, 1.1236% (2)

[61] Parietal_Inf_L, 0.561798% (1)

[65] Angular_L, 0.561798% (1)

Cluster 8 -> Cluster Size (Voxels): 169; Cluster Size (mm^3): 4563

Peak Index: 18 44 37

Peak Coordinate (X Y Z): 39 3 36

Peak Intensity: 7.78672

Label Include:

AAL -> Peak Label at [2] Precentral_R

[2] Precentral_R, 68.0473% (115)

[18] Rolandic_Oper_R, 12.426% (21)

[8] Frontal_Mid_R, 8.87574% (15)

[12] Frontal_Inf_Oper_R, 8.87574% (15)

[58] Postcentral_R, 1.18343% (2)

[4] Frontal_Sup_R, 0.591716% (1)

Cluster 9 -> Cluster Size (Voxels): 123; Cluster Size (mm^3): 3321

Peak Index: 44 22 25

Peak Coordinate (X Y Z): -39 -63 0

Peak Intensity: 5.81536

Label Include:

AAL -> Peak Label at [51] Occipital_Mid_L

[51] Occipital_Mid_L, 53.6585% (66)

[53] Occipital_Inf_L, 18.6992% (23)

[85] Temporal_Mid_L, 13.8211% (17)

[55] Fusiform_L, 7.31707% (9)

[89] Temporal_Inf_L, 6.50407% (8)

Cluster 10 -> Cluster Size (Voxels): 111; Cluster Size (mm^3): 2997

Peak Index: 14 45 19

Peak Coordinate (X Y Z): 51 6 -18

Peak Intensity: 6.99736

Label Include:

AAL -> Peak Label at [88] Temporal_Pole_Mid_R

[84] Temporal_Pole_Sup_R, 40.5405% (45)

[88] Temporal_Pole_Mid_R, 24.3243% (27)

[86] Temporal_Mid_R, 18.018% (20)

[82] Temporal_Sup_R, 17.1171% (19)

Cluster 11 -> Cluster Size (Voxels): 98; Cluster Size (mm^3): 2646

Peak Index: 48 28 16

Peak Coordinate (X Y Z): -51 -45 -27

Peak Intensity: -7.2433

Label Include:

AAL -> Peak Label at [89] Temporal_Inf_L

[89] Temporal_Inf_L, 95.9184% (94)

[85] Temporal_Mid_L, 4.08163% (4)

Cluster 12 -> Cluster Size (Voxels): 92; Cluster Size (mm^3): 2484

Peak Index: 24 53 19

Peak Coordinate (X Y Z): 21 30 -18

Peak Intensity: 5.71491

Label Include:

AAL -> Peak Label at [6] Frontal_Sup_Orb_R

[16] Frontal_Inf_Orb_R, 67.3913% (62)

[6] Frontal_Sup_Orb_R, 16.3043% (15)

[14] Frontal_Inf_Tri_R, 13.0435% (12)

[10] Frontal_Mid_Orb_R, 3.26087% (3)

Cluster 13 -> Cluster Size (Voxels): 79; Cluster Size (mm^3): 2133

Peak Index: 12 31 28

Peak Coordinate (X Y Z): 57 -36 9

Peak Intensity: 5.71322

Label Include:

AAL -> Peak Label at [82] Temporal_Sup_R

[82] Temporal_Sup_R, 73.4177% (58)

[86] Temporal_Mid_R, 21.519% (17)

[64] SupraMarginal_R, 3.79747% (3)

[66] Angular_R, 1.26582% (1)

Cluster 14 -> Cluster Size (Voxels): 78; Cluster Size (mm^3): 2106

Peak Index: 47 45 29

Peak Coordinate (X Y Z): -48 6 12

Peak Intensity: 5.30459

Label Include:

AAL -> Peak Label at [17] Rolandic_Oper_L

[1] Precentral_L, 38.4615% (30)

[11] Frontal_Inf_Oper_L, 21.7949% (17)

[17] Rolandic_Oper_L, 20.5128% (16)

[13] Frontal_Inf_Tri_L, 16.6667% (13)

[29] Insula_L, 2.5641% (2)

Cluster 15 -> Cluster Size (Voxels): 74; Cluster Size (mm^3): 1998

Peak Index: 21 23 38

Peak Coordinate (X Y Z): 30 -60 39

Peak Intensity: 5.87313

Label Include:

AAL -> Peak Label at [52] Occipital_Mid_R

[60] Parietal_Sup_R, 47.2973% (35)

[66] Angular_R, 24.3243% (18)

[50] Occipital_Sup_R, 10.8108% (8)

[52] Occipital_Mid_R, 8.10811% (6)

[68] Precuneus_R, 8.10811% (6)

[62] Parietal_Inf_R, 1.35135% (1)

Cluster 16 -> Cluster Size (Voxels): 67; Cluster Size (mm^3): 1809

Peak Index: 16 29 43

Peak Coordinate (X Y Z): 45 -42 54

Peak Intensity: 5.1001

Label Include:

AAL -> Peak Label at [62] Parietal_Inf_R

[62] Parietal_Inf_R, 73.1343% (49)

[58] Postcentral_R, 20.8955% (14)

[60] Parietal_Sup_R, 2.98507% (2)

[64] SupraMarginal_R, 2.98507% (2)

Cluster 17 -> Cluster Size (Voxels): 65; Cluster Size (mm^3): 1755

Peak Index: 33 29 43

Peak Coordinate (X Y Z): -6 -42 54

Peak Intensity: 3.9835

Label Include:

AAL -> Peak Label at [33] Cingulum_Mid_L

[67] Precuneus_L, 29.2308% (19)

[69] Paracentral_Lobule_L, 21.5385% (14)

[33] Cingulum_Mid_L, 20% (13)

[68] Precuneus_R, 15.3846% (10)

[70] Paracentral_Lobule_R, 13.8462% (9)

Cluster 18 -> Cluster Size (Voxels): 59; Cluster Size (mm^3): 1593

Peak Index: 16 48 29

Peak Coordinate (X Y Z): 45 15 12

Peak Intensity: 6.81734

Label Include:

AAL -> Peak Label at [12] Frontal_Inf_Oper_R

[12] Frontal_Inf_Oper_R, 61.0169% (36)

[14] Frontal_Inf_Tri_R, 37.2881% (22)

[30] Insula_R, 1.69492% (1)

Cluster 19 -> Cluster Size (Voxels): 58; Cluster Size (mm^3): 1566

Peak Index: 42 17 21

Peak Coordinate (X Y Z): -33 -78 -12

Peak Intensity: 3.63453

Label Include:

AAL -> Peak Label at [55] Fusiform_L

[55] Fusiform_L, 43.1034% (25)

[53] Occipital_Inf_L, 34.4828% (20)

[43] Calcarine_L, 8.62069% (5)

[47] Lingual_L, 6.89655% (4)

[51] Occipital_Mid_L, 6.89655% (4)

Cluster 20 -> Cluster Size (Voxels): 58; Cluster Size (mm^3): 1566

Peak Index: 27 23 34

Peak Coordinate (X Y Z): 12 -60 27

Peak Intensity: 6.30739

Label Include:

AAL -> Peak Label at [68] Precuneus_R

[68] Precuneus_R, 81.0345% (47)

[46] Cuneus_R, 10.3448% (6)

[48] Lingual_R, 6.89655% (4)

[44] Calcarine_R, 1.72414% (1)

Cluster 21 -> Cluster Size (Voxels): 50; Cluster Size (mm^3): 1350

Peak Index: 27 35 36

Peak Coordinate (X Y Z): 12 -24 33

Peak Intensity: -5.89139

Label Include:

AAL -> Peak Label at [34] Cingulum_Mid_R

[34] Cingulum_Mid_R, 56% (28)

[33] Cingulum_Mid_L, 44% (22)

**4.SBA:**

**ROI 2:**

Cluster 1 -> Cluster Size (Voxels): 733; Cluster Size (mm^3): 19791

Peak Index: 34 55 24

Peak Coordinate (X Y Z): -9 36 -3

Peak Intensity: 9.24292

Label Include:

AAL -> Peak Label at [31] Cingulum_Ant_L

[31] Cingulum_Ant_L, 27.0123% (198)

[32] Cingulum_Ant_R, 13.9154% (102)

[23] Frontal_Sup_Medial_L, 10.9141% (80)

[4] Frontal_Sup_R, 8.86767% (65)

[24] Frontal_Sup_Medial_R, 7.23056% (53)

[25] Frontal_Med_Orb_L, 6.68486% (49)

[3] Frontal_Sup_L, 5.8663% (43)

[26] Frontal_Med_Orb_R, 5.72988% (42)

[8] Frontal_Mid_R, 5.04775% (37)

[27] Rectus_L, 4.36562% (32)

[21] Olfactory_L, 2.18281% (16)

[28] Rectus_R, 1.22783% (9)

[7] Frontal_Mid_L, 0.682128% (5)

[22] Olfactory_R, 0.272851% (2)

Cluster 2 -> Cluster Size (Voxels): 684; Cluster Size (mm^3): 18468

Peak Index: 33 28 27

Peak Coordinate (X Y Z): -6 -45 6

Peak Intensity: 5.91381

Label Include:

AAL -> Peak Label at [43] Calcarine_L

[67] Precuneus_L, 22.3684% (153)

[33] Cingulum_Mid_L, 17.1053% (117)

[68] Precuneus_R, 13.8889% (95)

[43] Calcarine_L, 10.3801% (71)

[48] Lingual_R, 8.33333% (57)

[35] Cingulum_Post_L, 7.60234% (52)

[44] Calcarine_R, 5.55556% (38)

[36] Cingulum_Post_R, 3.65497% (25)

[69] Paracentral_Lobule_L, 2.63158% (18)

[45] Cuneus_L, 2.48538% (17)

[47] Lingual_L, 2.19298% (15)

[34] Cingulum_Mid_R, 2.04678% (14)

[46] Cuneus_R, 1.02339% (7)

[70] Paracentral_Lobule_R, 0.730994% (5)

Cluster 3 -> Cluster Size (Voxels): 295; Cluster Size (mm^3): 7965

Peak Index: 35 54 42

Peak Coordinate (X Y Z): -12 33 51

Peak Intensity: 6.30986

Label Include:

AAL -> Peak Label at [3] Frontal_Sup_L

[3] Frontal_Sup_L, 53.8983% (159)

[7] Frontal_Mid_L, 34.2373% (101)

[23] Frontal_Sup_Medial_L, 11.5254% (34)

[19] Supp_Motor_Area_L, 0.338983% (1)

Cluster 4 -> Cluster Size (Voxels): 227; Cluster Size (mm^3): 6129

Peak Index: 47 22 32

Peak Coordinate (X Y Z): -48 -63 21

Peak Intensity: 5.40615

Label Include:

AAL -> Peak Label at [85] Temporal_Mid_L

[65] Angular_L, 36.1233% (82)

[85] Temporal_Mid_L, 35.2423% (80)

[51] Occipital_Mid_L, 27.7533% (63)

[61] Parietal_Inf_L, 0.881057% (2)

Cluster 5 -> Cluster Size (Voxels): 177; Cluster Size (mm^3): 4779

Peak Index: 15 25 32

Peak Coordinate (X Y Z): 48 -54 21

Peak Intensity: 5.15548

Label Include:

AAL -> Peak Label at [86] Temporal_Mid_R

[66] Angular_R, 47.4576% (84)

[86] Temporal_Mid_R, 36.1582% (64)

[82] Temporal_Sup_R, 14.1243% (25)

[62] Parietal_Inf_R, 2.25989% (4)

Cluster 6 -> Cluster Size (Voxels): 135; Cluster Size (mm^3): 3645

Peak Index: 46 34 30

Peak Coordinate (X Y Z): -45 -27 15

Peak Intensity: 4.33144

Label Include:

AAL -> Peak Label at [17] Rolandic_Oper_L

[81] Temporal_Sup_L, 77.037% (104)

[85] Temporal_Mid_L, 10.3704% (14)

[17] Rolandic_Oper_L, 7.40741% (10)

[29] Insula_L, 2.96296% (4)

[79] Heschl_L, 1.48148% (2)

[63] SupraMarginal_L, 0.740741% (1)

**ROI 4:**

Cluster 1 -> Cluster Size (Voxels): 156; Cluster Size (mm^3): 4212

Peak Index: 47 54 37

Peak Coordinate (X Y Z): -48 33 36

Peak Intensity: -8.63717

Label Include:

AAL -> Peak Label at [7] Frontal_Mid_L

[7] Frontal_Mid_L, 75% (117)

[13] Frontal_Inf_Tri_L, 19.2308% (30)

[3] Frontal_Sup_L, 5.12821% (8)

[11] Frontal_Inf_Oper_L, 0.641026% (1)

Cluster 2 -> Cluster Size (Voxels): 152; Cluster Size (mm^3): 4104

Peak Index: 26 24 38

Peak Coordinate (X Y Z): 15 -57 39

Peak Intensity: -6.18592

Label Include:

AAL -> Peak Label at [68] Precuneus_R

[67] Precuneus_L, 48.6842% (74)

[68] Precuneus_R, 48.6842% (74)

[46] Cuneus_R, 1.97368% (3)

[45] Cuneus_L, 0.657895% (1)

Cluster 3 -> Cluster Size (Voxels): 86; Cluster Size (mm^3): 2322

Peak Index: 47 30 38

Peak Coordinate (X Y Z): -48 -39 39

Peak Intensity: -5.49465

Label Include:

AAL -> Peak Label at [61] Parietal_Inf_L

[61] Parietal_Inf_L, 94.186% (81)

[65] Angular_L, 4.65116% (4)

[63] SupraMarginal_L, 1.16279% (1)

Cluster 4 -> Cluster Size (Voxels): 81; Cluster Size (mm^3): 2187

Peak Index: 18 55 40

Peak Coordinate (X Y Z): 39 36 45

Peak Intensity: -6.91476

Label Include:

AAL -> Peak Label at [8] Frontal_Mid_R

[8] Frontal_Mid_R, 91.358% (74)

[4] Frontal_Sup_R, 8.64198% (7)

Cluster 5 -> Cluster Size (Voxels): 50; Cluster Size (mm^3): 1350

Peak Index: 46 16 33

Peak Coordinate (X Y Z): -45 -81 24

Peak Intensity: -6.08406

Label Include:

AAL -> Peak Label at [51] Occipital_Mid_L

[51] Occipital_Mid_L, 94% (47)

[85] Temporal_Mid_L, 6% (3)

**Ⅲ. The following section presents the visualized results of each statistically significant metric after FDR correction.**

**1.ALFF**


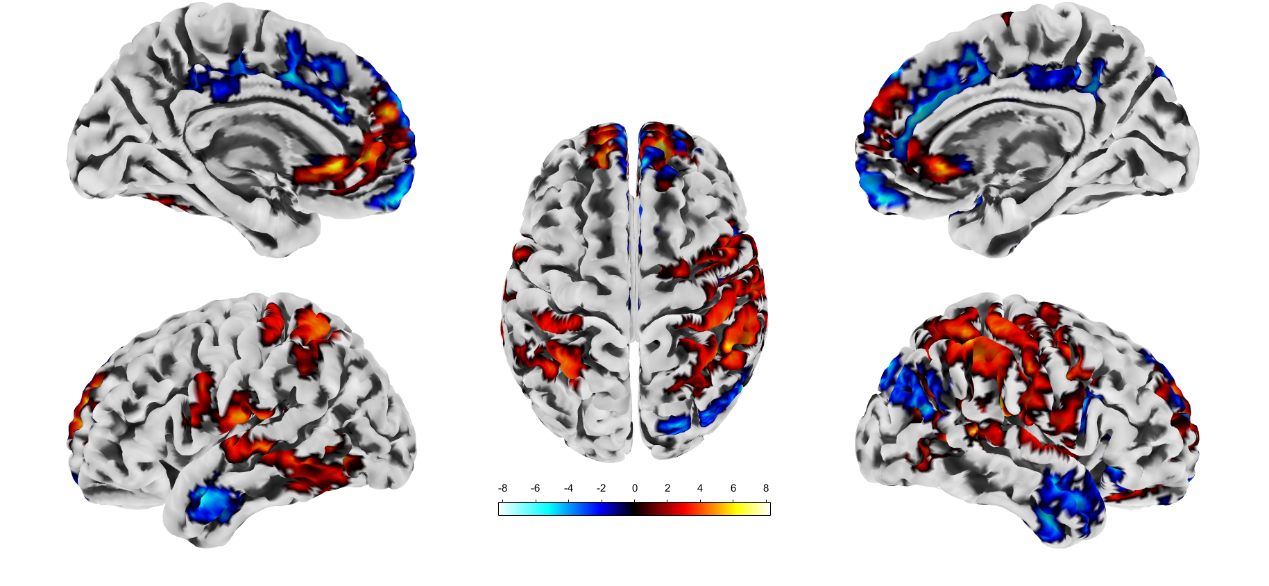


**2.fALFF:**


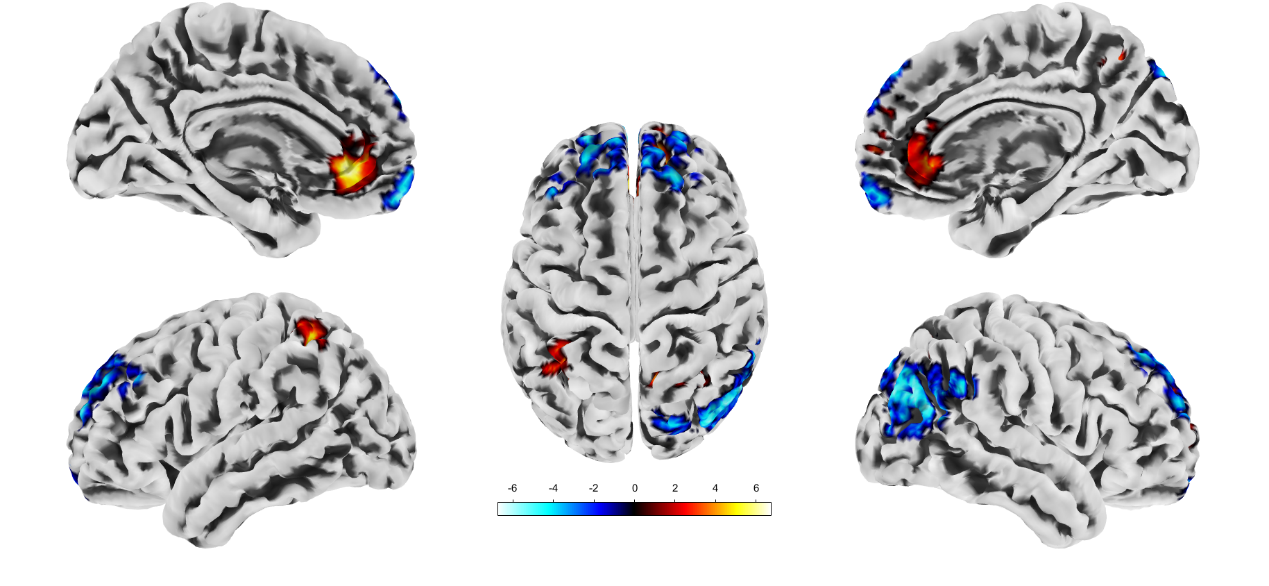


**3.Reho:**


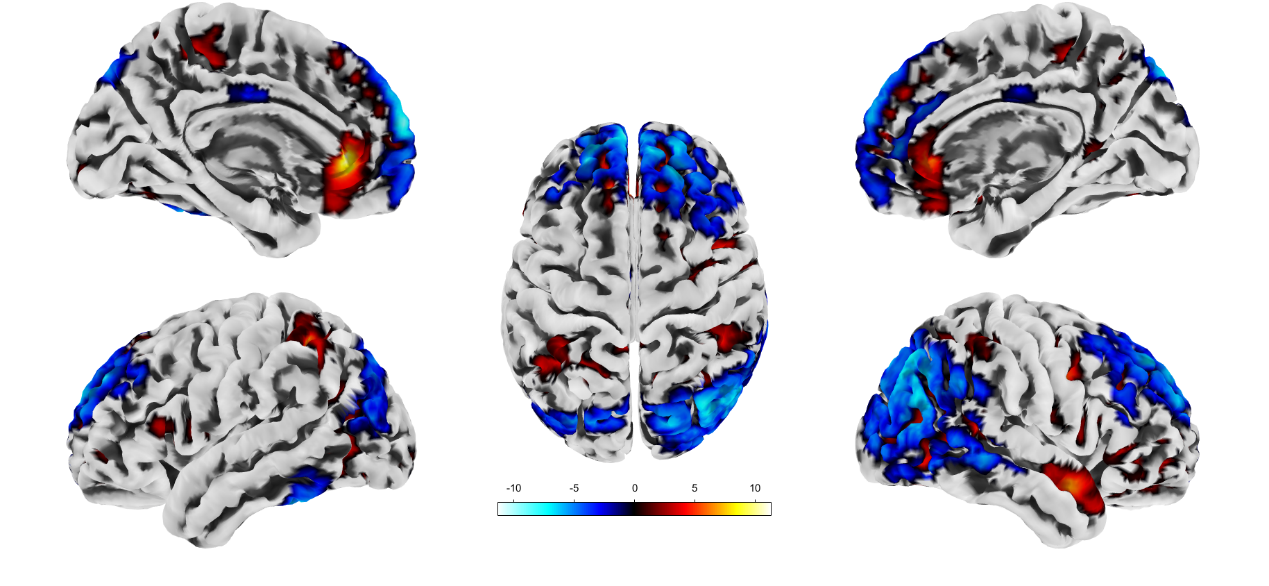


**4.SBA:**

**ROI 2:**


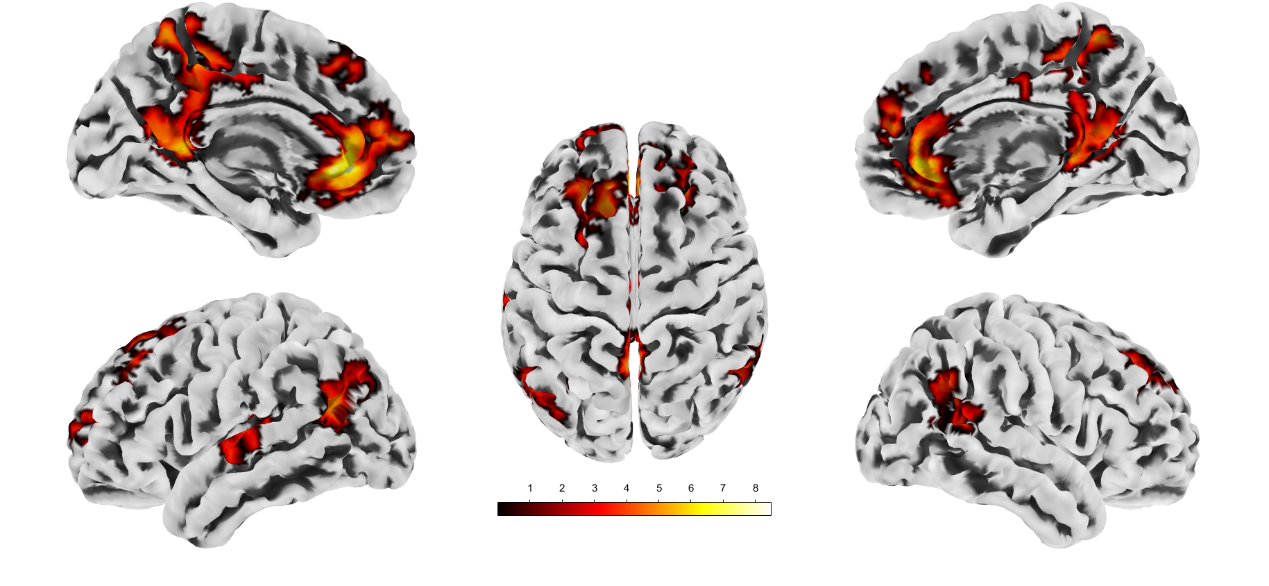


**ROI 4:**


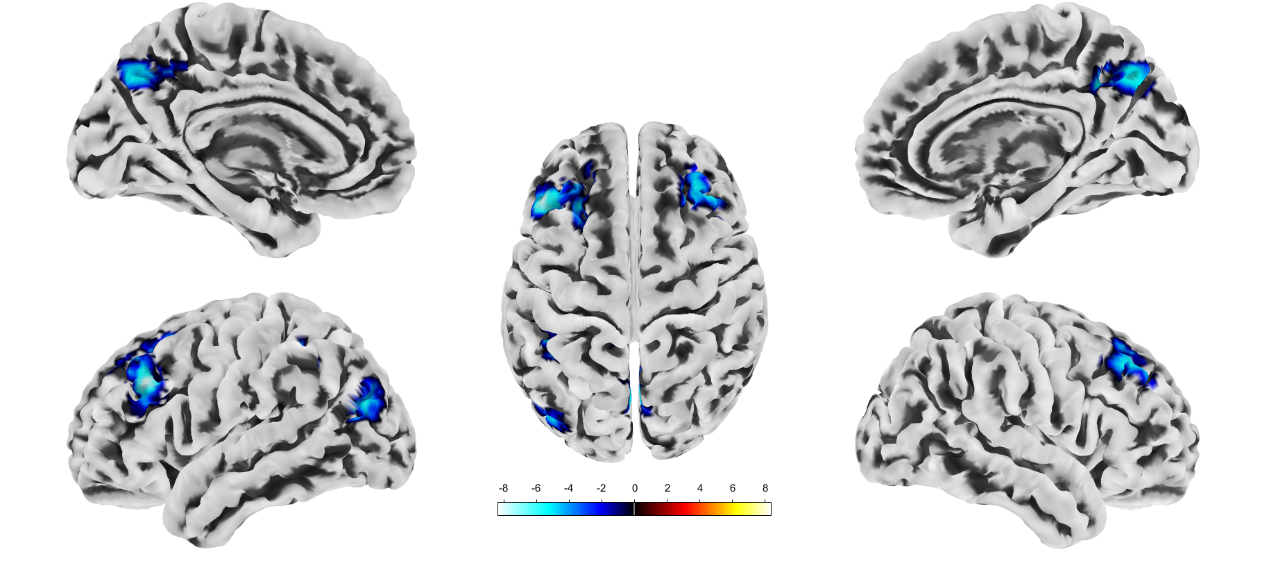

Supplement: Supplementary file 4 [file Supplementary_file_4.docx]
