# Supplementary material for: Cognitive and cortical network alterations in pediatric temporal lobe space-occupying lesions: an fMRI study
Source: Front Hum Neurosci. 2024 Dec 9;18:1509899. doi: 10.3389/fnhum.2024.1509899 (PMC11663916; doi:10.3389/fnhum.2024.1509899)
Supplement: Supplementary file 5 [file Supplementary_file_5.docx]

Supplementary Material 5：

Results of Spearman rank correlation analysis after FDR correction

| Items | Name of values | WM | SustA |
| --- | --- | --- | --- |
| ALFF_Cluster1 | correlation coefficient | -0.201 | -0.272 |
|  | Sig.(two-tails) | 0.304 | 0.162 |
|  | q_FDR | 0.57475 | 0.407906 |
| ALFF_Cluster2 | correlation coefficient | -0.088 | -0.094 |
|  | Sig.(two-tails) | 0.657 | 0.636 |
|  | q_FDR | 0.739882 | 0.659127 |
| ALFF_Cluster3 | correlation coefficient | -0.035 | -0.027 |
|  | Sig.(two-tails) | 0.860 | 0.893 |
|  | q_FDR | 0.875357 | 0.893 |
| ALFF_Cluster4 | correlation coefficient | 0.259 | 0.284 |
|  | Sig.(two-tails) | 0.183 | 0.143 |
|  | q_FDR | 0.57475 | 0.407906 |
| ALFF_Cluster5 | correlation coefficient | -0.149 | -0.219 |
|  | Sig.(two-tails) | 0.448 | 0.264 |
|  | q_FDR | 0.592023 | 0.418 |
| ALFF_Cluster6 | correlation coefficient | -0.299 | -0.254 |
|  | Sig.(two-tails) | 0.122 | 0.193 |
|  | q_FDR | 0.57475 | 0.407906 |
| ALFF_Cluster7 | correlation coefficient | 0.156 | 0.209 |
|  | Sig.(two-tails) | 0.428 | 0.287 |
|  | q_FDR | 0.592023 | 0.419462 |
| ALFF_Cluster8 | correlation coefficient | -0.333 | -0.349 |
|  | Sig.(two-tails) | 0.083 | 0.069 |
|  | q_FDR | 0.57475 | 0.407906 |
| ALFF_Cluster9 | correlation coefficient | -0.243 | -0.332 |
|  | Sig.(two-tails) | 0.212 | 0.084 |
|  | q_FDR | 0.57475 | 0.407906 |
| ALFF_Cluster10 | correlation coefficient | 0.276 | 0.355 |
|  | Sig.(two-tails) | 0.155 | 0.064 |
|  | q_FDR | 0.57475 | 0.407906 |
| ALFF_Cluster11 | correlation coefficient | -0.247 | -0.236 |
|  | Sig.(two-tails) | 0.205 | 0.226 |
|  | q_FDR | 0.57475 | 0.407906 |
| ALFF_Cluster12 | correlation coefficient | -0.183 | -0.282 |
|  | Sig.(two-tails) | 0.351 | 0.146 |
|  | q_FDR | 0.57475 | 0.407906 |
| ALFF_Cluster13 | correlation coefficient | 0.244 | 0.294 |
|  | Sig.(two-tails) | 0.211 | 0.129 |
|  | q_FDR | 0.57475 | 0.407906 |
| ALFF_Cluster14 | correlation coefficient | 0.206 | 0.209 |
|  | Sig.(two-tails) | 0.293 | 0.285 |
|  | q_FDR | 0.57475 | 0.419462 |
| ALFF_Cluster15 | correlation coefficient | -0.102 | -0.121 |
|  | Sig.(two-tails) | 0.606 | 0.541 |
|  | q_FDR | 0.719625 | 0.571056 |
| ALFF_Cluster16 | correlation coefficient | -0.207 | -0.235 |
|  | Sig.(two-tails) | 0.290 | 0.229 |
|  | q_FDR | 0.57475 | 0.407906 |
| ALFF_Cluster17 | correlation coefficient | -0.237 | -0.247 |
|  | Sig.(two-tails) | 0.226 | 0.205 |
|  | q_FDR | 0.57475 | 0.407906 |
| ALFF_Cluster18 | correlation coefficient | -0.123 | -0.188 |
|  | Sig.(two-tails) | 0.534 | 0.337 |
|  | q_FDR | 0.661696 | 0.458714 |
| fALFF_1 | correlation coefficient | -0.307 | -0.364 |
|  | Sig.(two-tails) | 0.112 | 0.057 |
|  | q_FDR | 0.57475 | 0.407906 |
| fALFF_2 | correlation coefficient | 0.141 | 0.181 |
|  | Sig.(two-tails) | 0.473 | 0.355 |
|  | q_FDR | 0.599133 | 0.459886 |
| fALFF_3 | correlation coefficient | 0.170 | 0.162 |
|  | Sig.(two-tails) | 0.386 | 0.411 |
|  | q_FDR | 0.5835 | 0.498447 |
| fALFF_4 | correlation coefficient | 0.179 | 0.241 |
|  | Sig.(two-tails) | 0.363 | 0.217 |
|  | q_FDR | 0.57475 | 0.407906 |
| fALFF_5 | correlation coefficient | -0.373 | -0.314 |
|  | Sig.(two-tails) | 0.051 | 0.103 |
|  | q_FDR | 0.57475 | 0.407906 |
| fALFF_6 | correlation coefficient | -0.245 | -0.315 |
|  | Sig.(two-tails) | 0.209 | 0.102 |
|  | q_FDR | 0.57475 | 0.407906 |
| fALFF_7 | correlation coefficient | 0.181 | 0.239 |
|  | Sig.(two-tails) | 0.356 | 0.221 |
|  | q_FDR | 0.57475 | 0.407906 |
| fALFF_8 | correlation coefficient | -0.275 | -0.303 |
|  | Sig.(two-tails) | 0.156 | 0.117 |
|  | q_FDR | 0.57475 | 0.407906 |
| fALFF_9 | correlation coefficient | -0.287 | -0.367 |
|  | Sig.(two-tails) | 0.139 | 0.054 |
|  | q_FDR | 0.57475 | 0.407906 |
| fALFF_10 | correlation coefficient | -0.078 | -0.144 |
|  | Sig.(two-tails) | 0.694 | 0.466 |
|  | q_FDR | 0.760731 | 0.533118 |
| fALFF_11 | correlation coefficient | -0.312 | -0.258 |
|  | Sig.(two-tails) | 0.106 | 0.184 |
|  | q_FDR | 0.57475 | 0.407906 |
| fALFF_12 | correlation coefficient | -0.321 | -0.292 |
|  | Sig.(two-tails) | 0.096 | 0.132 |
|  | q_FDR | 0.57475 | 0.407906 |
| Reho_Cluster_1 | correlation coefficient | 0.291 | 0.288 |
|  | Sig.(two-tails) | 0.133 | 0.138 |
|  | q_FDR | 0.57475 | 0.407906 |
| Reho_Cluster_2 | correlation coefficient | 0.170 | 0.223 |
|  | Sig.(two-tails) | 0.389 | 0.255 |
|  | q_FDR | 0.5835 | 0.415286 |
| Reho_Cluster_3 | correlation coefficient | -0.191 | -0.260 |
|  | Sig.(two-tails) | 0.330 | 0.181 |
|  | q_FDR | 0.57475 | 0.407906 |
| Reho_Cluster_4 | correlation coefficient | 0.182 | 0.185 |
|  | Sig.(two-tails) | 0.355 | 0.347 |
|  | q_FDR | 0.57475 | 0.459886 |
| Reho_Cluster_5 | correlation coefficient | -0.340 | -.433^*^ |
|  | Sig.(two-tails) | 0.077 | 0.021 |
|  | q_FDR | 0.57475 | 0.407906 |
| Reho_Cluster_6 | correlation coefficient | -0.272 | -0.359 |
|  | Sig.(two-tails) | 0.161 | 0.061 |
|  | q_FDR | 0.57475 | 0.407906 |
| Reho_Cluster_7 | correlation coefficient | -0.212 | -0.237 |
|  | Sig.(two-tails) | 0.280 | 0.224 |
|  | q_FDR | 0.57475 | 0.407906 |
| Reho_Cluster_8 | correlation coefficient | 0.196 | 0.247 |
|  | Sig.(two-tails) | 0.316 | 0.206 |
|  | q_FDR | 0.57475 | 0.407906 |
| Reho_Cluster_9 | correlation coefficient | -0.063 | -0.081 |
|  | Sig.(two-tails) | 0.749 | 0.681 |
|  | q_FDR | 0.776236 | 0.693161 |
| Reho_Cluster_10 | correlation coefficient | -0.147 | -0.201 |
|  | Sig.(two-tails) | 0.455 | 0.304 |
|  | q_FDR | 0.592023 | 0.4332 |
| Reho_Cluster_11 | correlation coefficient | 0.269 | 0.305 |
|  | Sig.(two-tails) | 0.166 | 0.115 |
|  | q_FDR | 0.57475 | 0.407906 |
| Reho_Cluster_12 | correlation coefficient | -0.086 | -0.140 |
|  | Sig.(two-tails) | 0.662 | 0.477 |
|  | q_FDR | 0.739882 | 0.533118 |
| ROI1_Cluster1 | correlation coefficient | -0.146 | -0.128 |
|  | Sig.(two-tails) | 0.457 | 0.516 |
|  | q_FDR | 0.592023 | 0.554943 |
| ROI1_Cluster2 | correlation coefficient | -0.108 | -0.141 |
|  | Sig.(two-tails) | 0.584 | 0.474 |
|  | q_FDR | 0.708255 | 0.533118 |
| ROI2_Cluster1 | correlation coefficient | -0.198 | -0.223 |
|  | Sig.(two-tails) | 0.312 | 0.254 |
|  | q_FDR | 0.57475 | 0.415286 |
| ROI2_Cluster2 | correlation coefficient | -.389^*^ | -.496^**^ |
|  | Sig.(two-tails) | 0.041 | 0.007 |
|  | q_FDR | 0.57475 | 0.399 |
| ROI2_Cluster3 | correlation coefficient | -0.064 | -0.212 |
|  | Sig.(two-tails) | 0.747 | 0.278 |
|  | q_FDR | 0.776236 | 0.419462 |
| ROI2_Cluster4 | correlation coefficient | -0.235 | -0.351 |
|  | Sig.(two-tails) | 0.229 | 0.067 |
|  | q_FDR | 0.57475 | 0.407906 |
| ROI2_Cluster5 | correlation coefficient | -0.195 | -0.335 |
|  | Sig.(two-tails) | 0.319 | 0.081 |
|  | q_FDR | 0.57475 | 0.407906 |
| ROI2_Cluster6 | correlation coefficient | -0.007 | -0.148 |
|  | Sig.(two-tails) | 0.972 | 0.452 |
|  | q_FDR | 0.972 | 0.533118 |
| ROI2_Cluster7 | correlation coefficient | -0.160 | -0.188 |
|  | Sig.(two-tails) | 0.415 | 0.338 |
|  | q_FDR | 0.592023 | 0.458714 |
| ROI4_Cluster1 | correlation coefficient | 0.256 | 0.245 |
|  | Sig.(two-tails) | 0.188 | 0.210 |
|  | q_FDR | 0.57475 | 0.407906 |
| ROI4_Cluster2 | correlation coefficient | 0.204 | 0.225 |
|  | Sig.(two-tails) | 0.297 | 0.250 |
|  | q_FDR | 0.57475 | 0.415286 |
| ROI4_Cluster3 | correlation coefficient | 0.154 | 0.137 |
|  | Sig.(two-tails) | 0.433 | 0.487 |
|  | q_FDR | 0.592023 | 0.533827 |
| ROI4_Cluster4 | correlation coefficient | 0.192 | 0.262 |
|  | Sig.(two-tails) | 0.328 | 0.179 |
|  | q_FDR | 0.57475 | 0.407906 |
| ROI4_Cluster5 | correlation coefficient | 0.095 | 0.163 |
|  | Sig.(two-tails) | 0.631 | 0.408 |
|  | q_FDR | 0.73402 | 0.498447 |
| ROI4_Cluster6 | correlation coefficient | 0.068 | 0.166 |
|  | Sig.(two-tails) | 0.733 | 0.400 |
|  | q_FDR | 0.776236 | 0.498447 |

Sig.(two-tails) means the p value before FDR correction

q_FDR means the p value corrected by FDR
